# Neuronal Excitability in the Medial Habenula and Ventral Tegmental Area Is Differentially Modulated by Nicotine Dosage and Menthol in a Sex-Specific Manner

**DOI:** 10.1523/ENEURO.0380-23.2024

**Published:** 2024-02-09

**Authors:** Nathan A. Olszewski, Samuel Tetteh-Quarshie, Brandon J. Henderson

**Affiliations:** Department of Biomedical Science and Research, Joan C. Edwards School of Medicine, Marshall University, Huntington 25703-1104, West Virginia

**Keywords:** electrophysiology, medial habenula, nicotine, nicotinic receptor, self-administration

## Abstract

The medial habenula (MHb) has been identified as the limiting factor for nicotine intake and facilitating nicotine withdrawal. However, few studies have assessed MHb neuronal excitability in response to nicotine, and, currently, a gap in knowledge is present for finding behavioral correlates to neuronal excitability in the region. Moreover, no study to date has evaluated sex or nicotine dosage as factors of excitability in the MHb. Here, we utilized an e-vape self-administration (EVSA) model to determine differences between sexes with different nicotine dosages ± menthol. Following this paradigm, we employed patch-clamp electrophysiology to assess key metrics of MHb neuronal excitability in relation to behavioral endpoints. We observed female mice self-administered significantly more than males, regardless of dosage. We also observed a direct correlation between self-administration behavior and MHb excitability with low-dose nicotine + menthol in males. Conversely, a high dose of nicotine ± menthol yields an inverse correlation between excitability and self-administration behavior in males only. In addition, intrinsic excitability in the ventral tegmental area (VTA) does not track with the amount of nicotine self-administered. Rather, they correlate to the active/inactive discrimination of mice. Using fast-scan cyclic voltammetry, we also observed that dopamine release dynamics are linked to reinforcement-related behavior in males and motivation-related behaviors in females. These results point to a sex-specific difference in the activity of the MHb and VTA leading to distinct differences in self-administration behavior. His could lend evidence to clinical observations of smoking and nicotine-use behavior differing between males and females.

## Significance Statement

Nicotine dependence remains a priority topic due to the fact that hundreds of thousands of individuals die annually due to tobacco-related illnesses. While the mesolimbic reward pathway has been proven to be critical to nicotine's actions, over time other brain areas have been identified to play crucial roles in nicotine reward, reinforcement, and withdrawal. One area, the medial habenula has increasingly been shown to be highly important. Here, our data suggest that the dopamine system of the mesolimbic pathway may be highly important for the learning aspect of nicotine intake, while the medial habenula may play a larger role in the amount of nicotine that is taken in a preclinical self-administration model.

## Introduction

The medial habenula (MHb) has been extensively studied as a key hub for negative emotions and negative prediction error (Salas et al., 2010; Zhang et al., 2016) and mood disorders such as anxiety (Yamaguchi et al., 2013) and depression (Han et al., 2017; Antunes et al., 2022). Additionally, the MHb plays a key role in the reinforcement of opioids (Taraschenko et al., 2007; Boulos et al., 2020) and cocaine (López et al., 2019) but is perhaps most associated with nicotine dependence due to its dense populations of nicotinic acetylcholine receptors (nAChRs) (Sheffield et al., 2000; Shih et al., 2014). Common among these are α3 (McCallum et al., 2012; Elayouby et al., 2021), α4 (Srinivasan et al., 2011), α5 (Fowler et al., 2011), α6 (Berry et al., 2015), β2 (Picciotto et al., 1998), and β4 (Salas et al., 2004; McCallum et al., 2012) subunit-containing nAChRs. The MHb is vital for both limiting nicotine intake (Fowler et al., 2011; Elayouby et al., 2021) and nicotine withdrawal-like symptoms (Baldwin et al., 2011; Zhao-Shea et al., 2015; Pang et al., 2016; Klenowski et al., 2022). The MHb projects almost exclusively to the interpeduncular nucleus (IPN) (Lima et al., 2017; McLaughlin et al., 2017) through glutamatergic and cholinergic projections (Ren et al., 2011; Wallace et al., 2020). The major downstream target of the MHb→IPN pathway is the laterodorsal tegmentum (LDTg) (McLaughlin et al., 2017; Quina et al., 2017) which modulates dopaminergic firing in the VTA (Lodge and Grace, 2006) and, thus, impacts dopaminergic-mediated reward (Lammel et al., 2012; Steidl et al., 2017). Further, the IPN→LDTg→VTA circuit has also been documented as facilitating nicotine aversion and avoidance through GABAergic projections from the IPN to the LDTg (Wolfman et al., 2018). Optogenetic inactivation of the MHb during nicotine withdrawal alleviates many withdrawal- and anxiety-like symptoms while significantly decreasing c-FOS-positive cells in the IPN (Zhao-Shea et al., 2015). Additionally, a “gain-of-function” α4 nAChR model in MHb cholinergic neurons produced increased anxiety, where antagonism of α4β2- and α6-containing nAChRs in the MHb attenuated anxiety (Pang et al., 2016). The MHb can further be divided into subregions. While most MHb neurons contain α3β4 nAChRs (Quick et al., 1999), the subregions are further characterized by distinct nAChR populations and gene expression (Aizawa et al., 2012; Wallace et al., 2020); with the lateral portion (lMHb) containing α4 nAChRs and the medial portion (mMHb) containing α6 nAChRs (Shih et al., 2014). These subregions all then project to distinct subregions of the IPN (Shih et al., 2014; Quina et al., 2017).

Previous studies documented the impact of acute (Dao et al., 2014; Lee et al., 2018) and chronic (Dao et al., 2014; Arvin et al., 2019) nicotine on MHb excitability. Dao et al. demonstrated that acute or long-term nicotine increased the firing rate of medial habenular neurons (Dao et al., 2014). Arvin and colleagues drew similar conclusions, demonstrating that chronic nicotine exposure increased the intrinsic excitability of MHb neurons (Arvin et al., 2019). Finally, Lee et al. showed contrasting inherent firing frequencies between subregions of the MHb, which persisted after acute application of nicotine in ex vivo brain slices (Lee et al., 2018). However, the limitations of these studies are that they do not account for variations in nicotine self-administration behavior between individuals. Noncontingent nicotine paradigms were utilized for all of the mentioned studies (Dao et al., 2014; Lee et al., 2018; Arvin et al., 2019). Thus, this study aimed to understand the link between differences in nicotine self-administration and neuronal excitability in the medial MHb. Additionally, we sought to determine the impact of menthol on the excitability of MHb neurons. Menthol remains one of the most common additives in tobacco and vaping products, which has been shown to both enhance the rewarding effects of nicotine (Henderson et al., 2017) and increase self-administration behavior (Cooper et al., 2021).

## Materials and Methods

### Animals

Experiments were conducted in accordance with guidelines for the use and care of animals provided by the National Institutes of Health. Experimental protocols were approved by the Institutional Animal Care and Use Committee at Marshall University. Adult (3–5-month-old) male and female C57BL/6J mice were acquired from in-house breeding colonies (wild-type, α6-GFPα4mCherry, or α6-GFP mice) or from Jackson laboratory. A total of 99 mice were used in the study. A total of 65 mice were used in the EVSA paradigm and medial habenular electrophysiology experiments (7 females and 8 males for 6 mg/ml nicotine, 9 females and 10 males for 6 mg/ml nicotine plus 15 mg/ml menthol, 8 females and 6 males for 60 mg/ml nicotine, and 8 females and 9 males for 60 mg/ml nicotine plus 15 mg/ml menthol). Additionally, 10 mice (5 females and 5 males) were used as control mice for electrophysiological experimentation. Finally, 8 male and 6 female α6-GFP mice were used in electrophysiology of the VTA, while 10 mice (5 male and 5 females) were used in FSCV experiments of the NAc. Preliminary and unpublished data from our laboratory have revealed no changes in self-administration behavior based on genotype. Mice were housed in a 12/12 h light/dark cycle and food and water were available *ad libitum*. Behavioral assessments were conducted during the light cycle.

### Drugs

Nicotine ditartrate dihydrate was obtained from Acros Organics (AC415660500). L-menthol [(−)-menthol] was obtained from Alfa Aesar (A10474). All nicotine and menthol formulations were made with propylene glycol and vegetable glycerin (PGVG) in a 50:50 ratio. Concentrations tested were PGVG alone, 6 mg/ml nicotine, 6 mg/ml nicotine + 15 mg/ml menthol, 60 mg/ml nicotine, and 60 mg/ml nicotine + 15 mg/ml menthol. Dose concentrations were chosen as they are based on previous experiments from our laboratory (Cooper et al., 2021; Henderson and Cooper, 2021) and closely mimic formulations found in commercial tank and pod-based vape liquids (Omaiye et al., 2019a, b).

### E-vape self-administration (EVSA)

We used a vapor self-administration setup (www.ljari.tech) previously utilized by our lab (Cooper et al., 2021, 2023b; Henderson and Cooper, 2021; Henderson et al., 2022). The operant self-administration setup used air-tight chambers with dimensions of 21 cm L × 19 cm W × 12.5 cm H [La Jolla Alcohol Research, Inc. (LJARI)]. The back of each chamber housed two Med Associates nose pokes with cue lights which were mounted above the floor. Passive chambers (www.ljari.tech) had the same interior dimensions as operant self-administration chambers but lacked nose pokes in the back of the chambers. Electric pumps created a vacuum-controlled airflow which was held at 1 L/min. Crown IV tanks with 0.4 Ω dual coils (UWELL, Ltd.) were activated by e-cigarette mod boxes (LJARI). Vapor delivery was controlled by e-vape custom controllers at 400°F and 65 W (LJARI) which equates to delivering ∼0.09 ml of e-liquid for every 3 s vape.

Mice began the paradigm on a Monday and for 5 d (Monday to Friday), were subjected to 2 h daily of noncontingent exposure to their treatment (groups mentioned previously). Groups with a nicotine dosage of 6 mg/ml ± menthol received a vapor delivery every 4 min and 48 s which totaled 25 vapor deliveries over 2 h. Groups assigned to a dosage of 60 mg/ml nicotine ± menthol received a vapor delivery every 12 min totaling 10 vapor deliveries in 2 h. These time periods for passive exposure were selected to expose mice to similar concentrations of nicotine yet limited in vapor delivery time (60 mg/ml nicotine ± menthol) to avoid toxic levels of nicotine being introduced which could induce stroke in mice. Following a week of acclimation, mice began EVSA in week 2. For the second and third weeks, mice were on a fixed ratio 1 (FR1) schedule for daily 2 h sessions for 10 d (Monday to Friday). For the entirety of the paradigm, there were weekend breaks between Friday and Monday sessions. Starting with FR1, during experimental sessions mice were singly housed in operant chambers. An active nose poke resulted in a 3 s vapor delivery, after which, a 30 s timeout period began and triggered a cue light in the nose poke. After the completion of the 10 FR1 sessions, mice were transitioned to an FR3 schedule in which three active pokes were then necessary to provide a vapor delivery. The FR3 schedule occurs for five sessions (11–15) during the fourth week of the paradigm. Week 5, and the final week of the paradigm, consisted of progressive ratio for sessions 16–18 where the FR criteria increased linearly for each vapor delivery. The final two sessions (19–20) were FR3. Previously, our lab has used a discrimination index of 2:1 active/inactive pokes during FR1 or FR3 to determine which mice acquire self-administration behavior (Cooper et al., 2021; Henderson and Cooper, 2021; Avelar et al., 2022; Henderson et al., 2022). Male mice reached the active: inactive nose poke criteria at a rate of 45% whereas females reached the criteria at a rate of 42%. However, this study sought to understand a relationship between all nicotine self-administration behavior and subsequent changes in neuronal excitability. For that reason, no mice were excluded from behavioral results.

### Patch-clamp electrophysiology

Brain slices from male and female mice that completed the above-described EVSA paradigm plus nicotine naive control animals having not undergone the paradigm (*n* = 5 males and *n* = 5 females) were used for electrophysiological recordings. All mice having undergone the EVSA paradigm were prepared for electrophysiological experimentation no more than 1 d following the last EVSA session. Following EVSA, mice were anesthetized with CO_2_. Mice were then subjected to a cardiac perfusion with NMDG artificial cerebrospinal fluid (NMDG-ACSF) saturated with 95% O_2_/5% CO_2_. NMDG-ACSF contained 93 mM NMDG, 2.5 mM KCl, 1.2 mM NaH_2_PO_4_, 1.2 mN NaHCO_3_, 20 mM HEPES, 25 mM Glucose, 5 mM Na-ascorbate, 2 mM Thiourea, 3 mM Na-pyruvate, 10 mM MgSO_4_·7H_2_O, and 0.5 mM CaCl_2_·2H_2_O. NMDG-ACSF was set to a pH of 7.2–7.4 with 10 N HCL and mOsm of 300–320 with sucrose. Brains were removed and set in agarose gel for slicing with a Compresstome VF-300-OZ (Precisionary Instruments). Coronal brain slices (300 µm) were sliced into ice-cold carbogenated NMDG-ACSF and slices containing the MHb (target bregma −1.4 to −1.8, Allen Brain Atlas) or VTA (target bregma −2.8 to −3.2) were collected and recovered at 37°C in NMDG-ACSF for 10 min. Slices were then transferred to standard ACSF containing 125 mM NaCl, 1.6 mM KCl, 1.2 mM NaH_2_PO_4_, 18 mM NaHCO_3_, 11 mM Glucose, 2.4 mM CaCl_2_, and 1.2 mM MgCl_2_. ACSF was set to pH 7.2–7.4 with 10 N HCL and mOSM of 300–320 with sucrose. Slices were allowed to recover in ACSF for 1 h at 37°C. After the hour of recovery, slices were transferred to the recording chamber and perfused with carbogenated ACSF (1.5–2.0 ml/min).

Neurons in the ventral tegmental area (VTA) or medial portion of the MHb (Shih et al., 2014) were visualized under an upright microscope (Axio Examiner A1, Zeiss) with an attached Axiocam 702 mono using DIC-IR 40x objective. Cell-attached and whole-cell recordings were obtained with an integrated patch-clamp amplifier (Sutter) abiding by our previous studies (Avelar et al., 2019; Akers et al., 2020; Cooper et al., 2023b). Borosilicate glass (outer diameter, 1.5 mm; inner diameter, 0.86 mm; Sutter Instrument) patch electrodes had resistances of 3–10 MΩ when filled with intrapipette solutionof the following: 135 mM K gluconate, 5 mM KCL, 5 mM EGTA, 0.5 mM CaCl_2_, 10 mM HEPES, 2 mM Mg-ATP, and 0.1 mM GTP. Recordings were sampled at ≥10 kHz. The junction potential between the bath solution and pipette was nulled before forming a gigaseal. For cell-attached voltage-clamp recordings, neurons were clamped at −65 mV and recorded for 60 s. For whole-cell current-clamp recordings, after whole-cell configuration was achieved, neurons were recorded for >3 min while being held in current clamp mode at 0 pA to allow for proper transfer of intrapipette solution. During this period, frequency of action potential firing (if any) was recorded. For control mice, action potential duration and threshold potential were obtained from these recordings. After this period, a current step protocol of −20 to +70 pA (5 pA steps) was first used to identify the rheobase of a neuron (minimal current necessary to elicit first action potential). Following this, a second step protocol of −100 to +700 pA (20 pA steps) was employed to find the maximal spiking ability of a neuron. For control animals, input resistance was calculated by averaging voltage responses from −100 and −60 pA hyperpolarizing currents and reported in GΩ which provided outputs like those previously reported (Dao et al., 2014).

### Fast-scan cyclic voltammetry (FSCV)

Brain slices including the NAc core (target bregma +1.0 mm; anterior–posterior limits of +1.4 to +0.7 mm; Allen Brain Atlas) were collected for FSCV using methods identical as electrophysiology assays. After recovery, slices were transferred to the recording chamber and a carbon fiber microelectrode was lowered to the NAc core. A 2 kHz triangular waveform (−0.4 V to +1.2 V and back to −0.4 V, at a rate of 400 V/s) was applied at 20 Hz (Demon Voltammetry). Dopamine release was stimulated (350 µA, Master-9) with a bipolar electrode (Plastics One), placed ∼250 µm from the recording electrode (100–250 µm away). Tonic-like stimulation was elicited with a five-pulse train at 5 Hz (0.2 ms inter-pulse interval, 1 s total stimulation time). Phasic-like stimulation was elicited with a five-pulse train at 60 Hz (16 ms inter-pulse intervals, 83 ms total stimulation time). Electrical stimulation was delivered at 3 min inter-stimulus intervals to avoid signal rundown. Electrodes were calibrated using 0, 0.01, 0.1, 1, and 10 µm dopamine standards. A total of eight mice were used (4 males and 4 females). To minimize the number of mice used, some of the above-listed mice were the same mice used in VTA electrophysiological assays.

### Statistical analysis

All results are presented as mean ± SEM and all statistical analyses were performed using GraphPad Prism 9.1.2. EVSA behavior with entire sessions (FR1, FR3, PR) and active nose pokes were analyzed via a two-way ANOVA (with Bonferroni's multiple comparison test) with sex and session as factors. Comparisons in FR3 nose pokes between treatment groups were meaned for all animals in the treatment group separately for sessions 11–15 and analyzed via a two-way ANOVA (with Bonferroni's multiple comparison test). Comparisons in PR nose pokes (breakpoint) were performed in the same manner except for sessions 16–18. For electrophysiology, differences in control male and female action potential duration, threshold potential, cell-attached firing frequency, whole-cell action potential frequency, rheobase, and maximum spikes per neuron were analyzed with unpaired *t*-tests. Plots of current/voltage relationship between control male and female MHb neurons were analyzed with mixed effects, two-way ANOVA (with Bonferroni's multiple comparison test) with injected pA and sex as factors. Correlations between FR3 score (mean nose pokes in sessions 11–15) and cell-attached firing frequency, whole-cell action potential frequency, rheobase, and maximum spikes were analyzed with simple linear regression. Additional correlations were performed between PR score (mean nose pokes in sessions 16–18) and the above-mentioned excitability metrics and were also analyzed via simple linear regression. All correlations are presented as the mean of values (±SEM) per excitability metric for each animal. Maximum spikes are presented as the highest spikes, whether in −20 to 70 pA step protocol or −100 to 700 pA step protocol. Differences in rheobase and maximal spiking ability between controls and nicotine dosage conditions (Fig. 7) were analyzed via one-way ANOVA with *post hoc* Tukey test.

## Results

### Male mMHb neurons show increased evoked excitability compared to females

**Figure 1.**
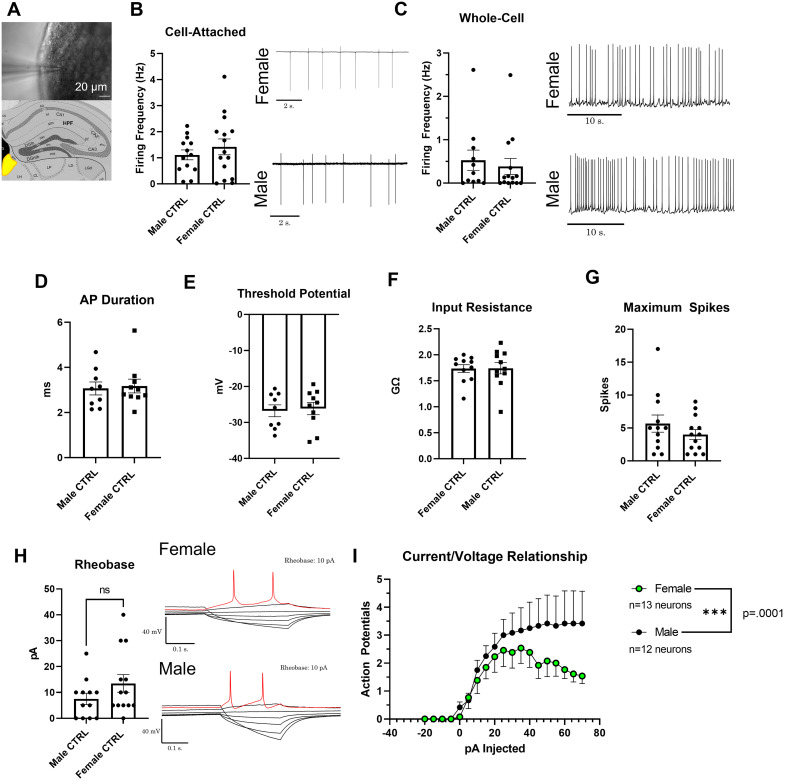
***A***, Schematic of target MHb region and DIC image of target MHb neurons. Scale bar, 20 µm. ***B***, Firing frequency mMHb neurons from PGVG-treated male and female mice in cell-attached mode. ***C***, Whole-cell firing frequency with representative traces for male and female mMHb neurons (*n* = 5 females (15 neurons cell-attached, 14 neurons whole-cell), *n* = 5 males (13 neurons cell-attached, 11 neurons whole-cell)). ***D***, Action potential duration, (***E***) threshold potential of mMHb neurons, (***F***) input resistance of control, and (***G***) maximal spiking ability of control mMHb neurons. ***H***, Rheobase (minimal current necessary to elicit first action potential) of control mMHb neurons found from −20 to +70 (5 pA steps) current step protocol with representative traces from females and males. Red traces represent the first elicited action potentials. ***I***, Current/voltage relationship plot of control mMHb neurons. *X*-axis represents currents injected from −20 to +70 pA and *Y*-axis is elicited action potentials per given current step. All results are mean ± SEM. Data represented as mean ± SEM were analyzed with an unpaired *t*-test (***B–H***) or mixed effects two-way ANOVA (***I***).

Prior to assessing the excitability of medial MHb (mMHb) neurons, we examined the intrinsic excitability of mMHb neurons between male and female mice naive to nicotine. As the MHb-IPN circuit may be a vital brain circuit for regulating nicotine intake (Fowler et al., 2011; Elayouby et al., 2021), understanding distinct differences between sexes is vital to understanding trends observed with nicotine intake. Utilizing patch-clamp electrophysiology in the mMHb (Fig. 1*A*), no sex differences were observed in firing frequency of mMHb neurons from control (no vapor exposure) mice in either cell-attached (Fig. 1*B*, *p* = 0.42, unpaired *t*-test) or whole-cell (Fig. 1*C*, *p* = 0.63, unpaired *t*-test) configurations. Additionally, membrane properties such as action potential duration (Fig. 1*D*, *p* = 0.808, unpaired *t*-test), threshold potential (Fig. 1*E*, *p* = 0.797, unpaired *t*-test), and input resistance (Fig. 1F*, p* = 0.956, unpaired *t*-test) showed no sex-dependent differences. In assessing intrinsic excitability, maximal spiking ability (Fig. 1*G*, *p* = 0.272, unpaired *t*-test), and rheobase (Fig. 1*H*, *p* = 0.083, unpaired *t*-test) showed no sex-dependent differences. It is important to note that the comparisons of rheobase between control male and female mice are trending towards significance with female mice showing an increased rheobase compared to males. Finally, there was a significant sex effect when intrinsic excitability was assessed with a current–voltage relationship [Fig. 1*I*, two-way ANOVA, *F*_(1,209) _= 15.3, *p *= 0.0001 (sex factor), *F*_(18,228) _= 7.5, *p* < 0.0001 (current injected factor); *F*_(18,209) _= 0.717, *p* = 0.792 (interaction)]. We observed that mMHb neurons in male mice reached a plateau of ∼4 action potentials; however, female mice peaked at ∼2 action potentials at 25 pA and decreased at increasing current steps.

### Female mice show increased self-administration behavior

**Figure 2.**
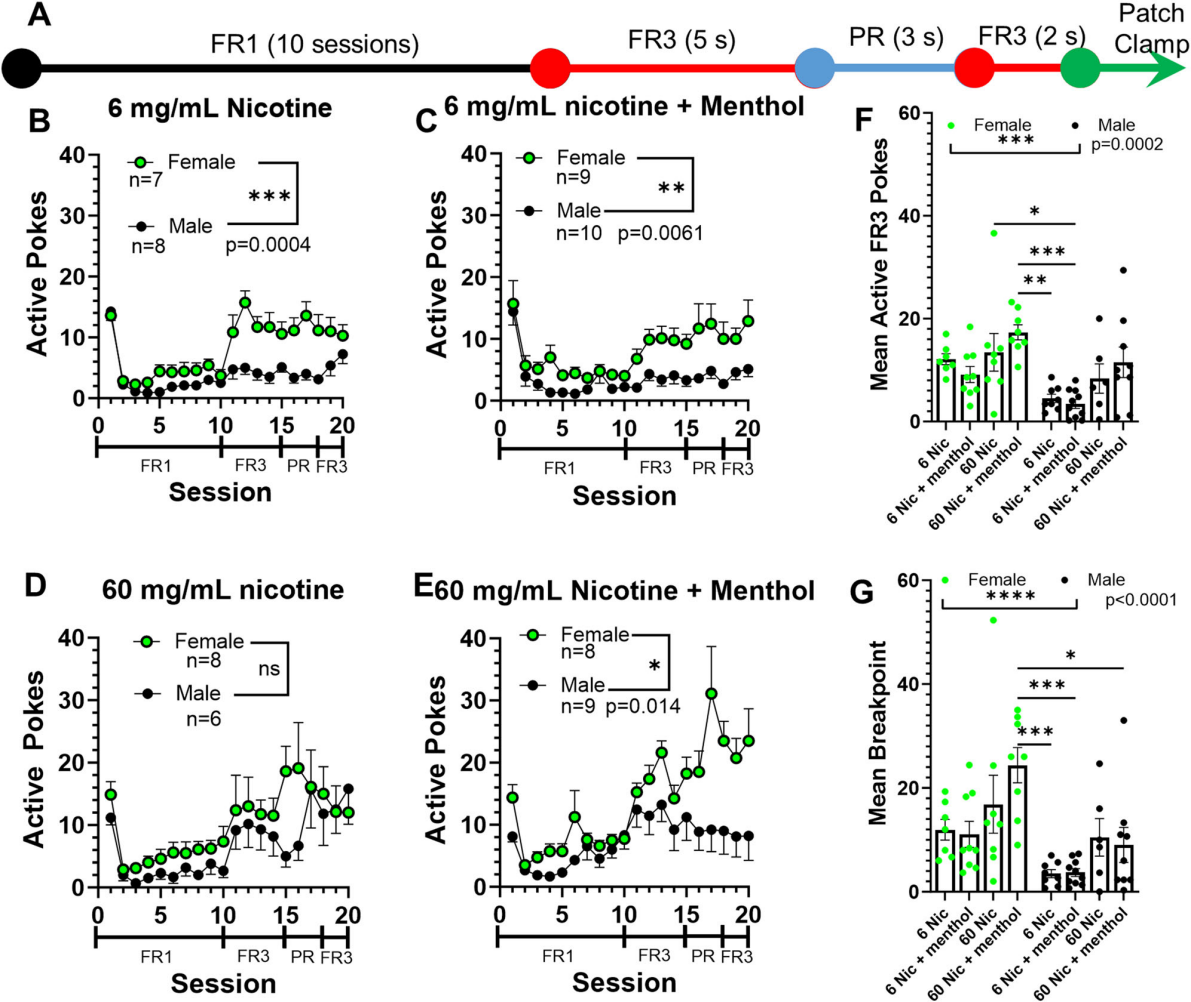
***A***, Timeline of EVSA paradigm. ***B–E***, Active nose pokes for male and female mice in our EVSA paradigm assigned to 6 mg/ml nicotine (***B***), 6 mg/ml nicotine plus menthol (***C***), 60 mg/ml nicotine (***D***), and 60 mg/ml nicotine plus menthol (***E***). ***F***, Mean FR3 active nose pokes for male and female mice. ***G***, Mean breakpoint for male and female mice. Data are presented as mean ± SEM and analyzed via two-way ANOVA. *p < 0.05; **p < 0.01; ***p < 0.001.

Using our established EVSA paradigm as we have previously documented (Henderson and Cooper, 2021; Cooper et al., 2023b), mice were allowed to self-administer in our 5-week-long EVSA paradigm (Fig. 2*A*) Here, male and female mice were assigned to 6 mg/ml nicotine, 6 mg/ml nicotine plus menthol, 60 mg/ml nicotine, or 60 mg/ml nicotine plus menthol (Fig. 2*B–E*). In previously published EVSA paradigms, mice were excluded if they did not achieve a 2:1 active-to-inactive nose poke distinction. Here, we did not exclude mice as we wanted to examine the neuronal function of mice that exhibited low nose poke behavior and failed to discriminate in addition to those that acquired self-administration behaviors.

Female mice assigned to 6 mg/ml nicotine [two-way ANOVA, *F*_(1,13) _= 21.83, *p* = 0.0004 (sex factor); *F*_(19,247) _= 15.45, *p *< 0.0001 (session factor); Fig. 2*B*], 6 mg/ml nicotine + 15 mg/ml menthol [two-way ANOVA, *F*_(1,17) _= 9.8, *p* = 0.0061 (sex factor); *F*_(19,323)_ = 10.07, *p *< 0.0001 (session factor); Fig. 2*C*], and 60 mg/ml nicotine + menthol (two-way ANOVA, *F*_(1,15)_ = 7.769, *p* = 0.014 (sex factor); *F*_(19,259)_ = 11.77, *p* < 0.0001 (session factor); Fig. 2*E*] all exhibited significantly more active nose pokes when compared to males. We did not observe a difference in EVSA between male and female mice with unflavored 60 mg/ml nicotine [two-way ANOVA, *F*_(1,12) _= 0.965, *p* = 0.345 (sex factor); *F*_(19,228) _= 7.368, *p* < 0.0001 (session factor); Fig. 2*D*]. To examine the effects of dose on reinforcement-related behavior in the FR3 period within sexes, we next looked at the mean active nose pokes for FR3 sessions 11–15 (Fig. 2*F*). There was a significant main effect with dose (*F*_(3,57) _= 5.9, *p* = 0.0014) and sex (*F*_(1,57) _= 16.02, *p* = 0.0002) with females showing significantly more FR3 nose pokes than male counterparts. Similarly, we examined the mean breakpoint for sessions 16–18 (Fig. 2*G*). Here, there was a significant main effect with dose (*F*_(3,57) _= 4.65, *p* = 0.0056) and sex (*F*_(1,57) _= 18.52, *p* < 0.0001).

### Female mMHb neuronal excitability only correlates to reinforcement-related behavior with high nicotine doses plus menthol

**Figure 3.**
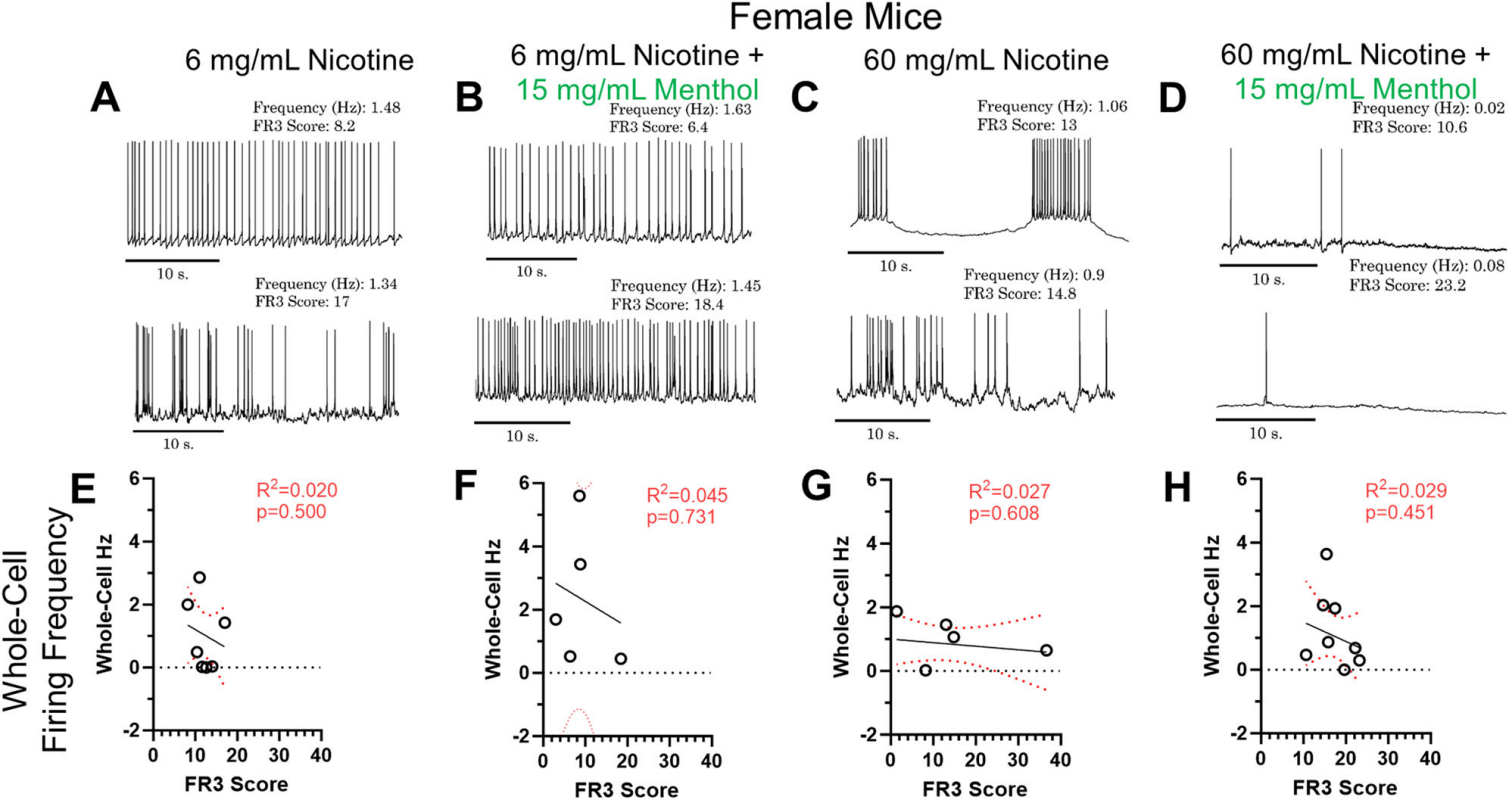
***A–D***, Representative current clamp baseline firing traces for 6 mg/ml nicotine (***A***), 6 mg/ml nicotine plus menthol (***B***), 60 mg/ml nicotine (***C***), and 60 mg/ml nicotine plus menthol (***D***). ***E–H***, Linear regression analysis of MHb baseline firing frequency to FR3 score for female mice assigned 6 mg/ml nicotine (***E***), 6 mg/ml nicotine plus menthol (***F***), 60 mg/ml nicotine (***G***), and 60 mg/ml nicotine plus menthol (***H***). Analysis was done through simple linear regression. Values for all animals are presented as mean per animal of two to five cells.

Recently, we showed that male and female mice that self-administer green apple chemical flavorants used in vaping products (in the absence of nicotine), exhibit an inverse correlation between self-administration behavior (active nose pokes and EVSA deliveries) and MHb intrinsic excitability (Cooper et al., 2023b). Similar to this previous investigation, we used female mice in our EVSA paradigm and upon completion examined the intrinsic excitability of mMHb neurons using brain slice patch-clamp electrophysiology (Fig. 3). We used three metrics to measure intrinsic excitability: (1) rheobase (the minimum current necessary to elicit an action potential), (2) maximum action potential spikes observed during current injections, and (3) baseline firing frequency.

For female mice assigned 6 mg/ml nicotine, 6 mg/ml nicotine plus menthol, and 60 mg/ml nicotine, we did not observe any correlation between FR3 active nose pokes and baseline whole-cell firing frequency (Fig. 3) for mice assigned to 6 mg/ml nicotine (*R*^2^ = 0.020, *p* = 0.50, Fig. 3*E*), 6 mg/ml nicotine plus menthol (*R*^2 ^= 0.045, *p* = 0.731, Fig. 3*F*), 60 mg/ml nicotine (*R*^2 ^= 0.027, *p* = 0.608, Fig. 3*G*), or 60 mg/ml nicotine plus menthol (*R*^2 ^= 0.029, *p* = 0.451, Fig. 3*H*).

**Figure 4.**
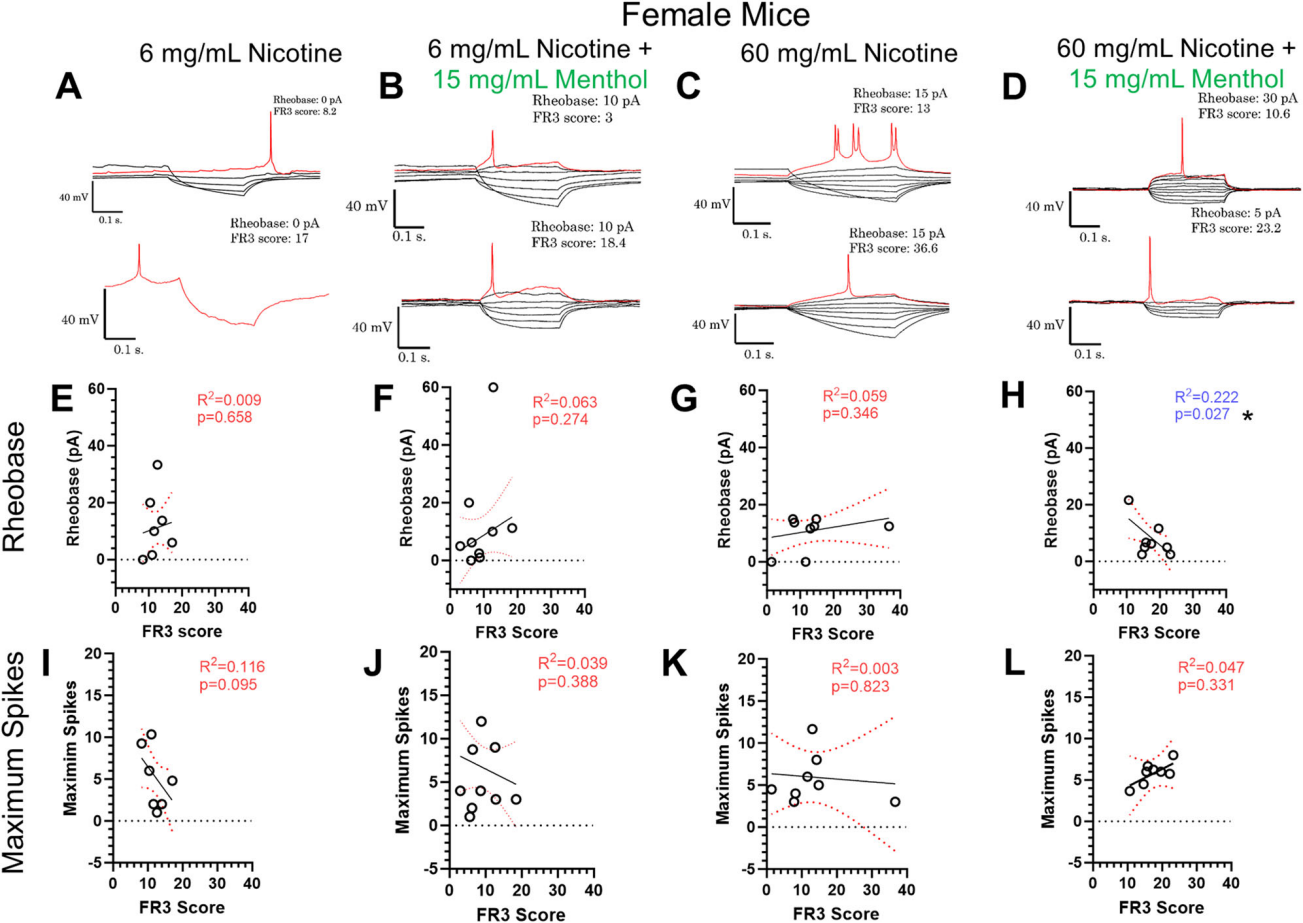
***A–D***, Representative current clamp traces of voltage steps for 6 mg/ml nicotine (***A***), 6 mg/ml nicotine plus menthol (***B***), 60 mg/ml nicotine (***C***), and 60 mg/ml nicotine plus menthol (***D***). ***E–H***, Linear regression analysis of rheobase to FR3 score for female mice assigned 6 mg/ml nicotine (***E***), 6 mg/ml nicotine plus menthol (***F***), 60 mg/ml nicotine (***G***), and 60 mg/ml nicotine plus menthol (***H***). ***I–L***, Linear regression analysis of maximum action potential spikes during current steps to FR3 score for female mice assigned 6 mg/ml nicotine (***I***), 6 mg/ml nicotine plus menthol (***J***), 60 mg/ml nicotine (***K***), and 60 mg/ml nicotine plus menthol (***L***). Values for all animals are presented as mean per animal. Blue *R*^2^ and *p* values with an asterisk(s) and red error bars represent significant correlations (*p* < 0.05). *N* = 6 mg/ml Nicotine, seven mice; 6 mg/ml nicotine plus menthol, nine mice; 60 mg/ml nicotine, eight mice; 60 mg/ml nicotine plus menthol: eight mice with two to five cells per animal.

Additionally, when assessing intrinsic excitability of female mice that underwent our EVSA paradigm with 6 mg/ml nicotine (Fig. 4*A*,*E*,*I*), there was no correlation between FR3 score and rheobase (*R*^2 ^= 0.009, *p* = 0.658, Fig. 4*E*) or maximum spikes (*R*^2 ^= 0.116, *p* = 0.095, Fig. 4*I*). Similar to these results, adding menthol (Fig. 4*B*,*F*,*J*) to this low dose of nicotine yielded no correlation between FR3 score and rheobase (*R*^2 ^= 0.063, *p* = 0.274, Fig. 3*F*) or maximum spikes (*R*^2 ^= 0.039, *p* = 0.388, Fig. 4*J*). Additionally, the higher dose of 60 mg/ml nicotine (Fig. 4*C*,*G*,*K*) in females also yielded no correlation between FR3 score and rheobase (*R*^2 ^= 0.059, *p* = 0.345, Fig. 4*G*) or maximum spikes (*R*^2 ^= 0.052, *p* = 0.823, Fig. 4*K*). However, for female mice assigned 60 mg/ml nicotine plus menthol (Fig. 4*D*,*H*,*L*), there was a significant direct correlation between FR3 score and rheobase (*R*^2 ^= 0.222, *p* = 0.027, Fig. 4*H*); but no correlation was observed between maximum spikes and FR3 score (*R*^2 ^= 0.047, *p* = 0.331, Fig. 4*L*).

Finally, correlates between PR score and neuronal excitability were assessed for female animals that completed the EVSA paradigm. No significant correlations were observed (Table 1) for any assigned dosages of e-liquid and any metric of excitability.

**Table 1.** Female progressive ratio correlations

	6 mg/ml nic	6 mg/ml + menthol	60 mg/ml nic	60 mg/ml + menthol
Whole-cell Hz	*R*^2 ^= 0.101 *p *= 0.121	*R*^2 ^= 0.008 *p *= 0.747	*R*^2 ^= 0.011 *p *= 0.75	*R*^2 ^= 0.019 *p *= 0.546
Rheobase	*R*^2 ^= 0.002 *p *= 0.845	*R*^2 ^= 0.000 *p *= 0.954	*R*^2 ^= 0.048 *p *= 0.397	*R*^2 ^= 0.065 *p *= 0.251
Max. spikes	*R*^2 ^= 0.149 *p *= 0.063	*R*^2 ^= 0.088 *p *= 0.191	*R*^2 ^= 0.009 *p *= 0.722	*R*^2 ^= 0.061 *p *= 0.269

Table of correlations for female mice with varying nicotine dosages (6 mg/ml nicotine, 6 mg/ml nicotine plus menthol, 60 mg/ml nicotine, 60 mg/ml nicotine plus menthol) between PR score and metrics of mMHb intrinsic excitability (whole-cell firing frequency, rheobase, maximum spikes). Represented are *R*^2^ and *p* values from the linear regressions.

### Both nicotine dose and presence of menthol differentially modulate medial habenular excitability in male mice only

**Figure 5.**
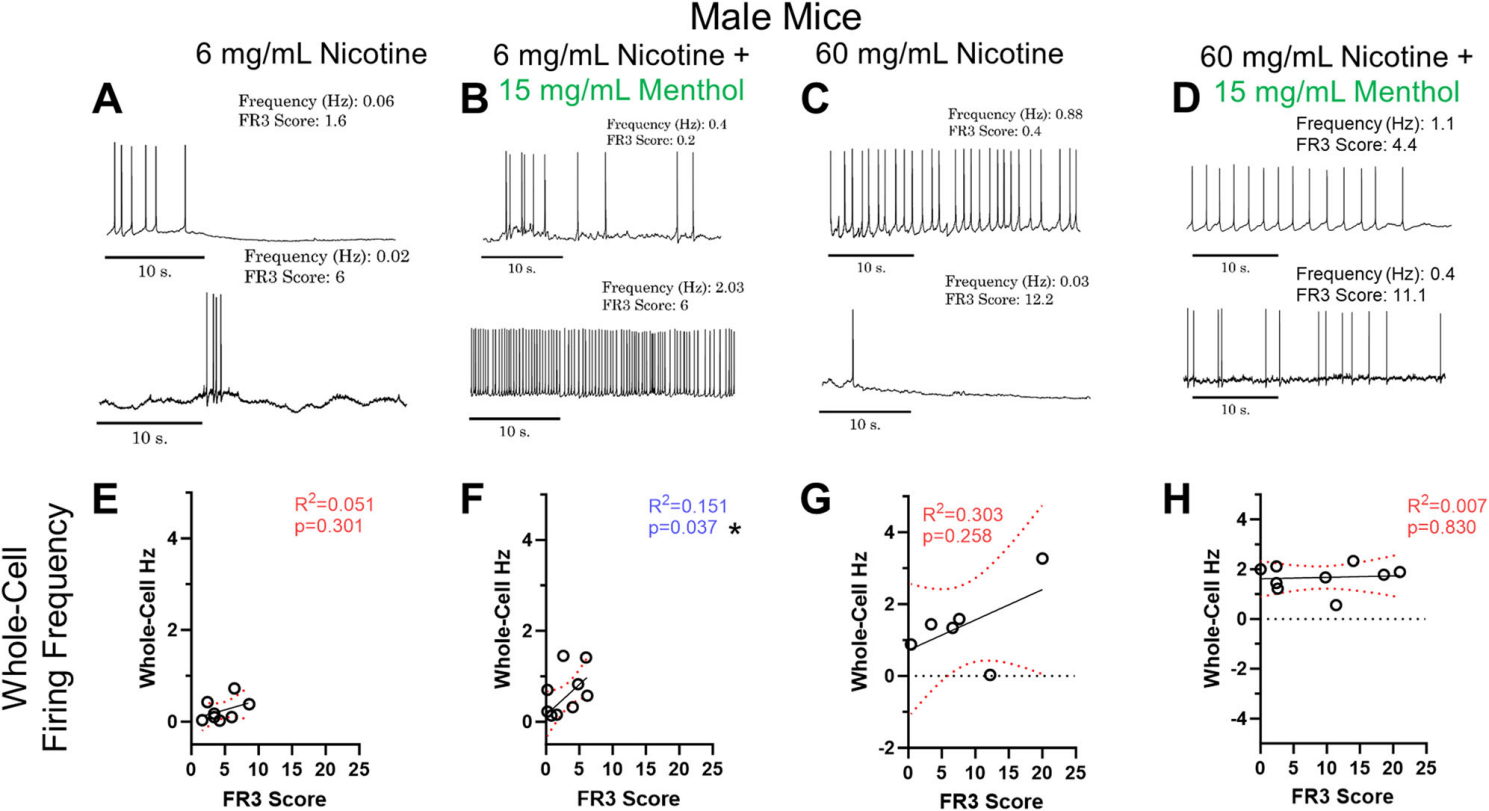
***A–D***, Representative current-clamp traces baseline firing traces for male mice assigned to 6 mg/ml nicotine (***A***), 6 mg/ml nicotine plus menthol (***B***), 60 mg/ml nicotine (***C***), and 60 mg/ml nicotine plus menthol (***D***). ***E–H***, Linear regression analysis of MHb baseline firing frequency to FR3 score for male mice assigned 6 mg/ml nicotine (***E***), 6 mg/ml nicotine plus menthol (***F***), 60 mg/ml nicotine (***G***), and 60 mg/ml nicotine plus menthol (***H***). Analysis was done through simple linear regression. Values for all animals are presented as mean per animal of 2–7 cells per animal. Blue *R*^2^ and *p* values with an asterisk(s) and red error bars represent significant correlations (*p* < 0.05).

Sex differences in nicotine self-administration have been documented to be highly variable depending on dosage (Jensen et al., 2016) with males preferring lower doses of nicotine (Cooper et al., 2021, 2023a) and displaying an aversion towards higher doses of nicotine (Isiegas et al., 2009). We first assessed the baseline firing frequency of male mice (Fig. 5) that had completed the EVSA paradigm. No significant correlation in firing frequency was observed for male mice assigned to 6 mg/ml nicotine (*R*^2 ^= 0.051, *p* = 0.301, Fig. 5*E*), 60 mg/ml nicotine (*R*^2 ^= 0.303, *p* = 0.258, Fig. 5*G*), or 60 mg/ml nicotine plus menthol (*R*^2 ^= 0.007, *p* = 0.83, Fig. 5*H*). However, a significant correlation was observed with male mice assigned to 6 mg/ml nicotine plus menthol (*R*^2 ^= 0.151, *p* = 0.037, Fig. 5*F*), meaning the as FR3 score increased, an increase in whole-cell firing frequency was observed.

**Figure 6.**
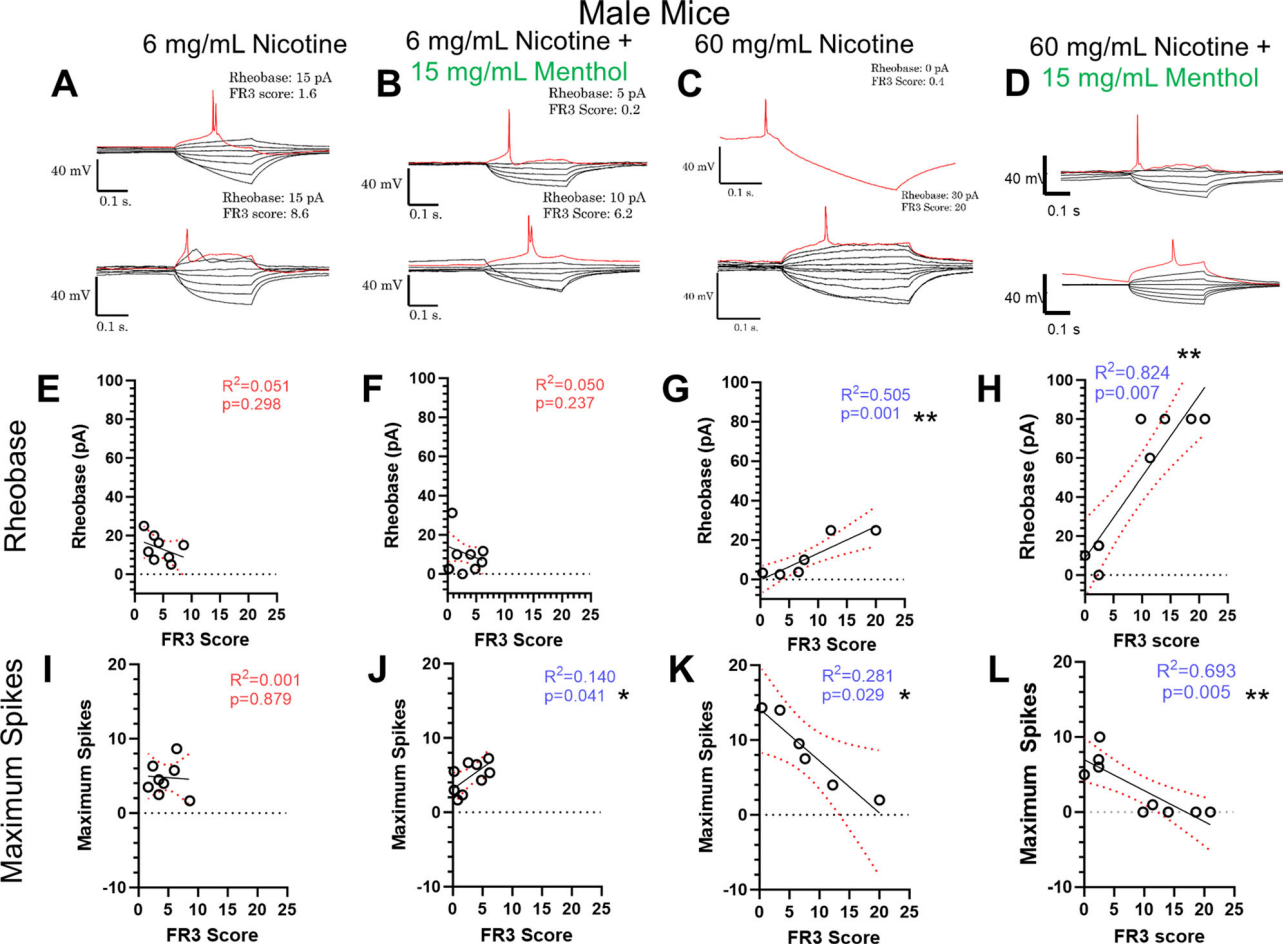
***A–D***, Representative current clamp traces of voltage steps for 6 mg/ml nicotine (***A***), 6 mg/ml nicotine plus menthol (***B***), 60 mg/ml nicotine (***C***), and 60 mg/ml nicotine plus menthol (***D***). ***E–H***, Linear regression analysis of rheobase to FR3 score for male mice assigned 6 mg/ml nicotine (***E***), 6 mg/ml nicotine plus menthol (***F***), 60 mg/ml nicotine (***G***), and 60 mg/ml nicotine plus menthol (***H***). ***I–L***, Linear regression analysis of maximum action potentials spikes during current steps to FR3 score for male mice assigned 6 mg/ml nicotine (***I***), 6 mg/ml nicotine plus menthol (***J***), 60 mg/ml nicotine (***K***), and 60 mg/ml nicotine plus menthol (***L***). Analysis was done through simple linear regression. Values for all animals are presented as mean. Blue *R*^2^ and *p* values with an asterisk(s) and red error bars represent significant correlations (*p* < 0.05). *N* = 6 mg/ml Nicotine, eight mice; 6 mg/ml nicotine plus menthol, nine mice; 60 mg/ml nicotine, six mice; 60 mg/ml nicotine plus menthol, nine mice with two to seven cells per animal.

When assessing intrinsic excitability metrics for male mice that completed the EVSA paradigm, male mice assigned 6 mg/ml nicotine e-liquids, showed no correlation between FR3 score and rheobase (*R*^2 ^= 0.0001, *p* = 0.879, Fig. 4*E*) or maximum spikes (*R*^2 ^= 0.051, *p* = 0.301, Fig. 6*I*).

For male mice assigned to 6 mg/ml nicotine + menthol (Fig. 6*B*,*F*,*J*), a direct correlation was observed between maximum spikes (*R*^2 ^= 0.140, *p* = 0.041, Fig. 6*J*) when compared to FR3 score. Accordingly, as the FR3 score increases, these mMHb neurons exhibited increased intrinsic excitability. No correlation was observed with rheobase (*R*^2 ^= 0.050, *p* = 0.237, Fig. 6*F*) for male mice having gone through the EVSA paradigm with 6 mg/ml nicotine + menthol. These results demonstrate a possible modulation of MHb excitability dependent on menthol with low doses of nicotine in males.

For male mice assigned 60 mg/ml nicotine, we observed a significant inverse correlation between FR3 score and rheobase (*R*^2 ^= 0.505, *p* = 0.0014, Fig. 6*G*). Additionally, there was a significant correlation between the FR3 score and maximum spikes (*R*^2 ^= 0.281, *p* = 0.029, Fig. 6*K*). For male mice assigned 60 mg/ml nicotine plus menthol, we observed a significant inverse correlation between FR3 score and rheobase (*R*^2 ^= 0.824, *p* = 0.007, Fig. 6*H*) and maximum spikes (*R*^2 ^= 0.693, *p* = 0.005, Fig. 6*L*). These results point to differential modulation of mMHb neuronal populations dependent on both presence of menthol (with low-dose nicotine) and nicotine dosage itself.

Finally, correlates between PR score and neuronal excitability were assessed for male animals that completed the EVSA paradigm. Generally, no significant correlations were observed (Table 2) for assigned dosages of e-liquid and any metric of excitability. However, significant correlations were observed with males assigned to 6 mg/ml nicotine (*R*^2 ^= 0.182, *p* = 0.042) and 6 mg/ml nicotine plus menthol (*R*^2 ^= 0.169, *p* = 0.027) when comparing PR score with whole-cell firing frequency.

**Table 2.** Male progressive ratio correlations

	6 mg/ml nic	6 mg/ml + menthol	60 mg/ml nic	60 mg/ml + menthol
Whole-cell Hz	*R*^2 ^= 0.182 *p *= 0.042	*R*^2 ^= 0.169 *p *= 0.027	*R*^2 ^= 0.072 *p *= 0.398	*R*^2 ^= 0.349 *p *= 0.094
Rheobase	*R*^2 ^= 0.01 *p *= 0.653	*R*^2 ^= 0.087 *p *= 0.113	*R*^2 ^= 0.137 *p *= 0.144	*R*^2 ^= 0.345 *p *= 0.097
Max. Spikes	*R*^2 ^= 0.000 *p *= 0.964	*R*^2 ^= 0.107 *p *= 0.083	*R*^2 ^= 0.092 *p *= 0.236	*R*^2 ^= 0.051 *p *= 0.559

Table of correlations for male mice with varying nicotine dosages (6 mg/ml nicotine, 6 mg/ml nicotine plus menthol, 60 mg/ml nicotine, 60 mg/ml nicotine plus menthol) between PR score and metrics of mMHb intrinsic excitability (whole-cell firing frequency, rheobase, maximum spikes). Represented are *R*^2^ and *p* values from the linear regressions. Green values represent significant correlations with *p *< 0.05.

### Nicotine dosage alters excitability of male, but not female, MHb neurons

**Figure 7.**
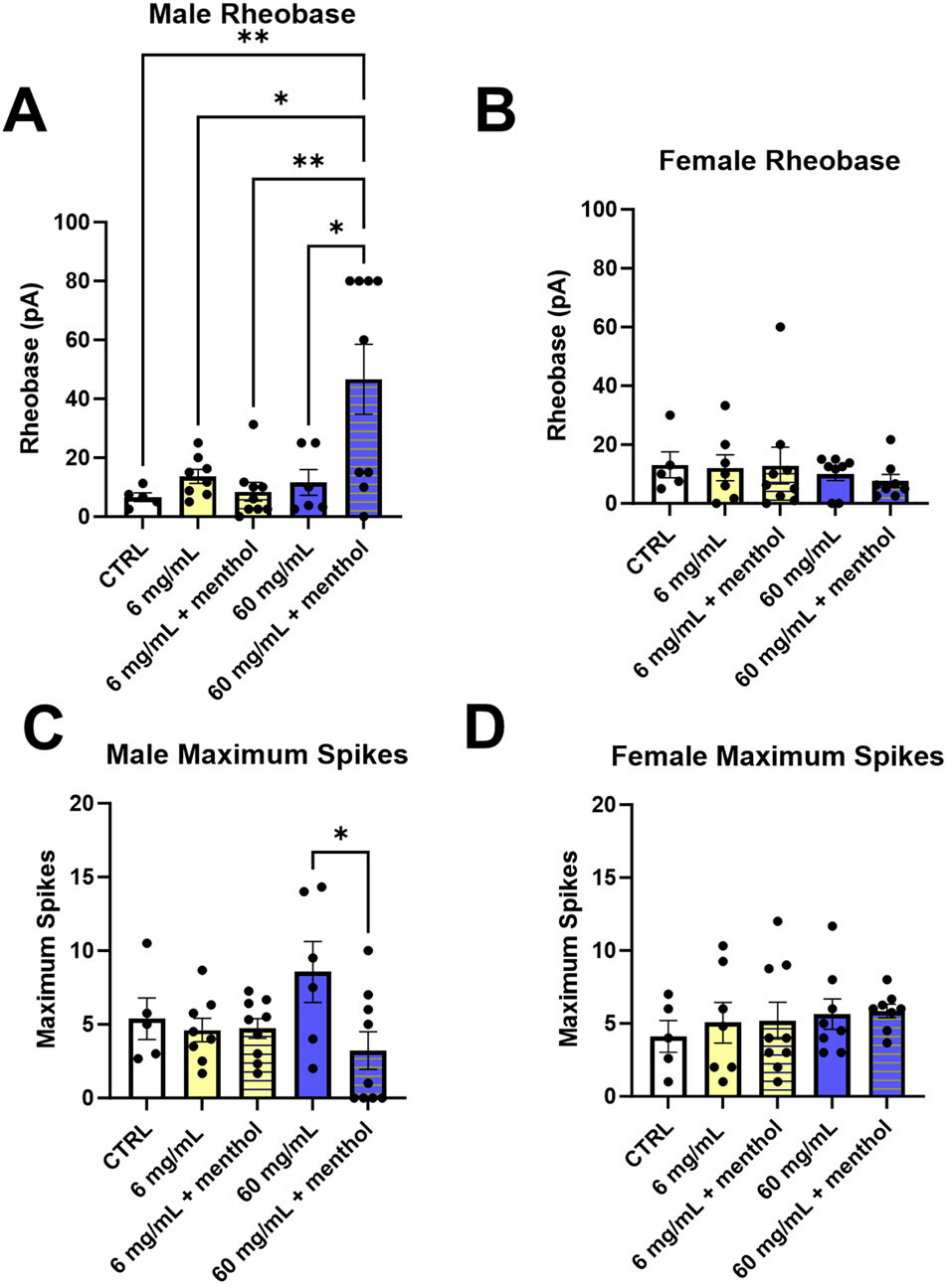
***A***, Comparison of rheobase between controls and tested nicotine dosages in male mice. ***B***, Comparison of rheobase between controls and tested nicotine dosages in female mice. ***C***, Comparison of maximal spiking ability between controls and tested nicotine dosages in male mice. ***D***, Comparison of maximal spiking ability between controls and tested nicotine dosages in female mice. Data points represent mean rheobase or maximal spiking ability per animal. Bar graphs represent mean ± SEM and were analyzed via one-way ANOVA. *p < 0.05; **p < 0.01.

We next sought to compare the excitability of MHb neurons between controls and mice that underwent the EVSA paradigm with varying nicotine dosages within sexes (Fig. 7*A–D*). Figure 7*A* shows differences in rheobase within male mice. A significant main effect was detected (*F* = 6.243, *p* = 0.0008) via one-way ANOVA. *Post hoc* Tukey analysis revealed a significant increase in rheobase between male mice that self-administered 60 mg/ml nicotine plus menthol and control mice (*p* = 0.006), 6 mg/ml mice (*p* = 0.010), 6 mg/ml plus menthol mice (*p* = 0.002), and 60 mg/ml nicotine mice (*p* = 0.012). Conversely, female mice (both controls and females that underwent the EVSA paradigm at all dosages) did not exhibit significant changes in rheobase (Fig. 7*B*).

Comparison of maximal spiking ability was also performed between controls and varying nicotine dosages for both males and females (Fig. 7*C*,*D*). For males, one-way ANOVA showed no significant main effect. However, *post hoc* Tukey analysis revealed a significant change in maximum spikes between 60 mg/ml nicotine and 60 mg/ml nicotine plus menthol mice (*p* = 0.035) with male mice that had self-administered 60 mg/ml nicotine only having a significantly larger maximal spiking ability (Fig. 7*C*). Conversely, and similar to the rheobase comparison, females showed no significant changes in maximal spiking ability across controls and all dosages of nicotine (Fig. 7*D*).

### VTA dopamine neuron excitability does not track with reinforcement-related behavior but it does with operant discrimination

While our data suggest the mMHb may be linked to reinforcement-related behavior of nicotine, we have previously determined that VTA dopamine neuron excitability is linked to reward-related behaviors (Akers et al., 2020). Additionally, we have previously observed that female mice prefer 60 mg/ml nicotine ± menthol (Cooper et al., 2023a). In this same report, we observed that males may prefer 6 mg/ml nicotine instead of 60 mg/ml nicotine. Additionally, males only exhibit a menthol-induced enhancement in reinforcement-related behavior with 6 mg/ml nictoine and not 60 mg/ml nicotine (Cooper et al., 2023a). We hypothesize that males assigned 6 mg/ml nicotine (plus menthol) and female mice assigned 60 mg/ml nicotine (plus menthol) exhibit a direct correlation between intrinsic excitability of mMHb neurons and reinforcement-related behaviors because these doses provide nicotine levels that are within a range that is reinforcing. For this reason, our final investigation into changes in VTA dopamine neuron intrinsic excitability used 6 mg/ml nicotine plus menthol for male mice but 60 mg/ml nicotine plus menthol for female mice.

**Figure 8.**
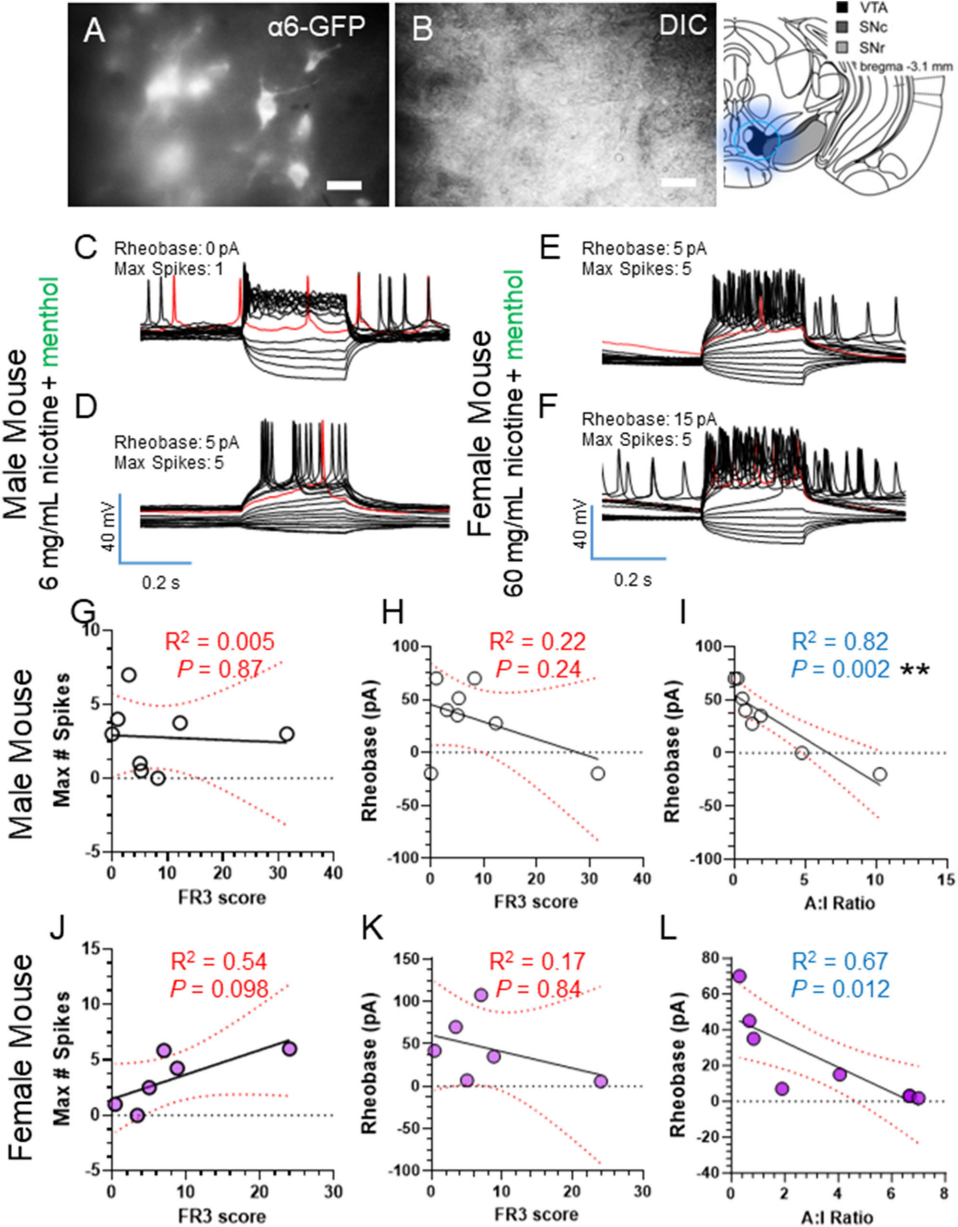
***A,B***, Representative UV and DIC images of α6-GFP neurons in the VTA. ***C–F***, Representative current clamp recordings of individual voltage steps for rheobase and maximum spikes from two individual VTA dopamine neurons. ***G–I***, Linear correlation of FR3 score with maximum action potential spike number (***G***), rheobase (***H***), and A:I ratio correlated to rheobase (***I***) for male mice. ***J–L***, Linear correlation of FR3 score with maximum action potential spike number (***J***), rheobase (***K***), and A:I ratio correlated to rheobase (***L***) for female mice. Data are means of two to three cells for eight male and six female mice (each dot is the mean of individual mice).

Using parallel cohorts of α6-GFP mice that went through the same EVSA paradigm as the mice used in the MHb assays, we collected VTA slices and identified putative dopamine neurons by α6-GFP fluorescence (Fig. 8*A*,*B*). Similar to the MHb assays, we used a current step protocol to measure intrinsic excitability by determining rheobase and maximum spikes within current steps. In both male and female mice, we observed that FR3 score did not correlate to maximum spikes or rheobase of VTA dopamine neurons (Fig. 8*G*,*H*,*J*,*K*). In addition to examining reinforcement-related behavior (FR3 score), we also examined the relationship between active and inactive distinction during the FR3 period (session 11–15). Accordingly, we calculated the mean of the active/inactive ratio for FR3 sessions (11–15) and correlated these values to VTA dopamine neuron intrinsic excitability. Here, we observed a significant correlation between active-to-inactive ratio and rheobase in male (*R*^2 ^= 0.82, *p* = 0.002, Fig. 8*I*) and female mice (*R*^2 ^= 0.67, *p* = 0.012, Fig. 8*L*).

**Figure 9.**
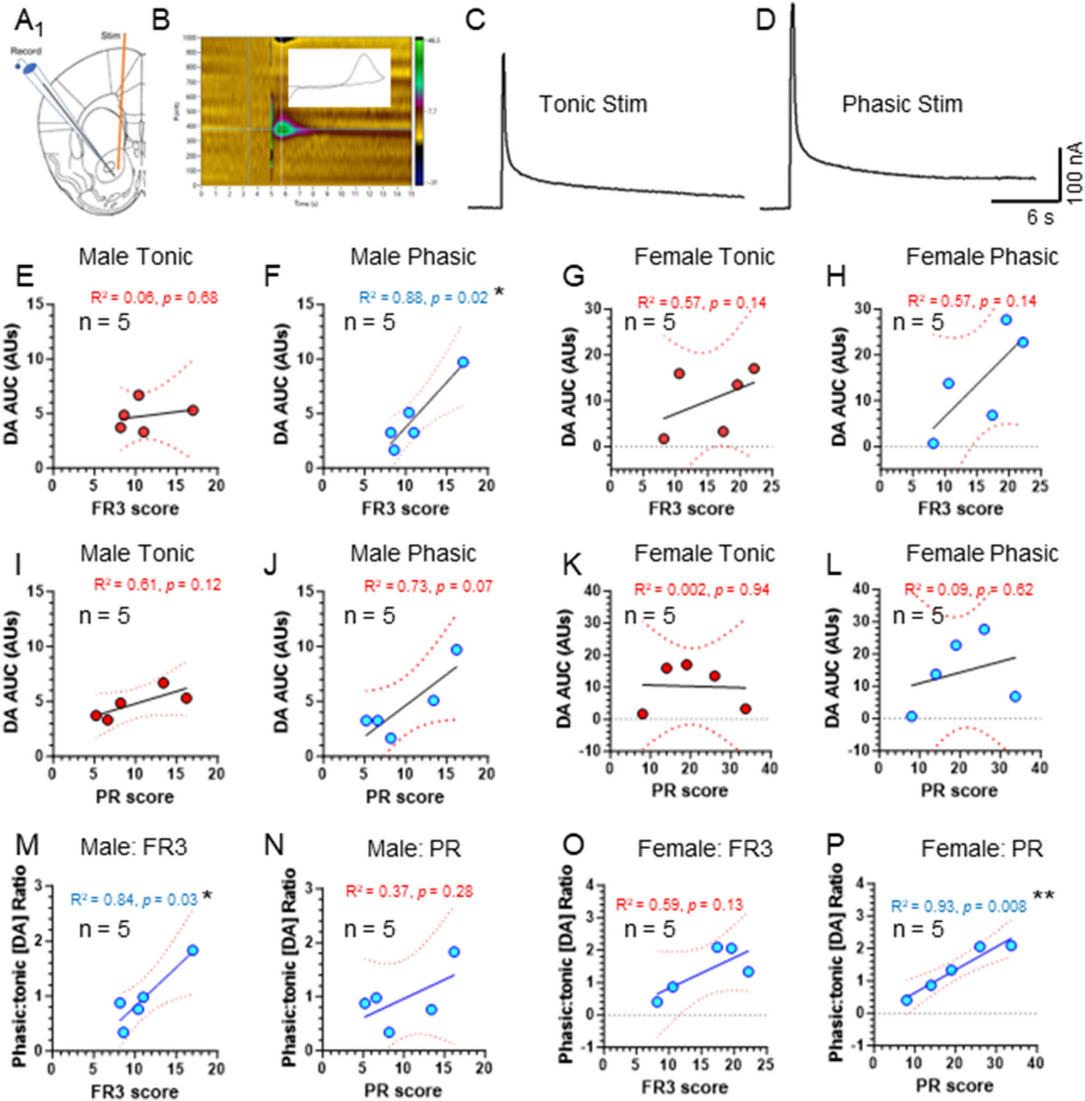
***A***, Schematic of recording electrode and stimulator placement for brain slice FSCV assays in the NAc core. ***B***, Representative voltammogram (insert) and color plot for a 60 Hz, 5-pulse stimulation from the NAc of a male mouse treated with nicotine plus menthol. ***E–H***, Linear regression of FR3 score (mean FR3 active nose pokes) to tonic and phasic DA release (area under the curve, AUC) for male (***E*,*F***) and female (***G*,*H***) mice. ***I–L***, Linear regression of PR score (mean breakpoint) to tonic and phasic DA release (area under the curve, AUC) for male (***I*,*J***) and female (***K*,*L***) mice. ***M–P***, Linear regression of FR3 score (mean FR3 active nose pokes) to phasic/tonic ratio for male (***M*,*N***) and female (***O*,*P***) mice. Each dot represents mean data from individual mice.

From these same mice, we collected brain slices that contain the nucleus accumbens (NAc) core and measured tonic (5 pulses, 5 Hz) and phasic (5 pulses, 60 Hz) dopamine release using fast-scan cyclic voltammetry (FSCV). Here, we correlated FR3 score and PR score to dopamine release in male and female mice (Fig. 9*E–H*,*I–L*). In male mice we observed a significant linear regression between phasic DA release and FR3 score (Fig. 9*F*, *R*^2 ^= 0.88, *p *= 0.02). We did not observe any significant correlations between FR3 or PR scores and NAc DA release in female mice. We also correlated FR3 score and PR score to phasic/tonic ratios calculated for individual mice. Here, we observed that male mice exhibited a significant correlation between phasic/tonic ratio and FR3 score (Fig. 9*M*, *R*^2 ^= 0.84, *p *= 0.03) and a significant correlation in female mice between phasic/tonic ratio and PR score (Fig. 9*P*, *R*^2 ^= 0.93, *p *= 0.008).

## Discussion

Our present study, first, utilized a nicotine vapor self-administration model to determine differences in self-administration between sexes as well as differences that nicotine dosages and menthol elicited. Next, we utilized patch-clamp electrophysiology and observed changes that occurred in mMHb excitability as a result of individual levels of self-administration. We observed distinct differences in self-administration in which females self-administered significantly more nicotine. Our electrophysiological assays then showed inherent differences in mMHb excitability between males and females. Finally, we showed contrasting correlations in mMHb excitability in males and females dependent on nicotine dosage and the presence of menthol. These results shed light on the dose- and sex-dependent activation of the mMHb and how this may play an important role in nicotine intake.

To our knowledge, this investigation is one of the first to identify distinct correlates between nicotine self-administration behavior (with or without menthol) and changes in mMHb neuronal excitability. Nicotine usage, and specifically the use of e-cigarettes and vaping products, continues to grow in popularity in all age groups (Schneller et al., 2018; Sapru et al., 2020; Boakye et al., 2022). Thus, it is vital to understand how vaping-related behaviors may be mediated by specific neuronal populations. The medial habenula is a region evolutionarily conserved in vertebrates (Stephenson-Jones et al., 2012). Further, clinical studies have demonstrated the crucial role of the MHb in drug dependence as postmortem analysis of human males addicted to heroin revealed a decrease in overall MHb volume and a decrease in total MHb neurons (Müller et al., 2021). Key findings in the field have shown that the MHb is crucial for facilitating nicotine intake and that genetic abnormalities in the CHRNA5/A3/B4 gene cluster (encoding for α5, α3, and β4 nAChRs, respectively), all of which are heavily populated in the MHb, can alter nicotine intake (Fowler et al., 2011; Icick et al., 2020; Elayouby et al., 2021). Further, elegant work by Zhao-Shea et al. has demonstrated that the MHb is vital for facilitating nicotine withdrawal, as optogenetic inhibition the MHb alleviated many of the somatic signs during a precipitated nicotine withdrawal (Zhao-Shea et al., 2015). Despite these important contributions, there have been no investigations into understanding how intrinsic excitability of the MHb corelates to nicotine intake.

Here, we observed that females self-administered significantly more than their male counterparts in most of the tested dosages (±menthol). This result may be, in part, surprising as higher dosages of nicotine have been shown to be aversive (Fowler and Kenny, 2014; Liu et al., 2022). However, sex-specific differences in nicotine self-administration have been thoroughly documented and previous studies have shown that females tend to self-administer more nicotine their than male counterparts (Donny et al., 2000; Rezvani et al., 2008; Bagdas et al., 2019; Flores et al., 2019; Cooper et al., 2023a). We observed a sex-specific difference between male and female nicotine-naive mMHb. Male mMHb neurons, in a current-voltage relationship, exhibited more action potential firing at higher currents (Fig. 1*I*). Conversely, female mMHb neurons reached a peak and then exhibited a decrease in action potential firing at higher current injections (Fig. 1*I*). Next, we documented that female mMHb neurons did not exhibit correlations between self-administration behavior and intrinsic excitability when assigned to 6 mg/ml nicotine, 6 mg/ml nicotine plus menthol, and 60 mg/ml nicotine. However, we did observe a significant inverse correlation between rheobase and FR3 score when female mice were assigned 60 mg/ml nicotine plus menthol (Fig. 4*H*). We have previously published a report that in comparing the doses of 6 and 60 mg/ml nicotine, female mice self-administer significantly more when assigned 60 mg/ml nicotine (Cooper et al., 2023a). Additionally, in this prevoius study we observed that menthol enhanced the self-administration of nicotine only at a dose of 60 mg/ml (Cooper et al., 2023a). Thus, the previously observed “preferred” dose for female mice is the only dose at which we observed a correlation between nicotine EVSA and mMHb intrinsic excitability. Moreover, the correlation we observed suggests that as female mice self-administer more nicotine (60 mg/ml) plus menthol, their mMHb neurons exhibit a higher intrinsic excitability (lower rheobase, more action potential firing).

We also observed that the intrinsic excitability of mMHb neurons from male mice were linked to reinforcement-related behaviors when assigned 6 mg/ml nicotine plus menthol, 60 mg/ml nicotine, or 60 mg/ml nicotine plus menthol. Here, we also noted that the correlation was differentially linked depending on the concentration of nicotine. The lower nicotine dose (6 mg/ml) plus menthol directly correlated with intrinsic excitability (maximum spikes and baseline firing frequency) and reinforcement-related behaviors. For the higher dose of nicotine (60 mg/ml) we observed an inverse correlation between mMHb intrinsic excitability and reinforcement-related behaviors. Thus, at the low dose plus menthol, mMHb neurons increased in intrinsic excitability as male mice self-administered more. However, at the high dose (with or without menthol), mMHb excitability decreased as male mice self-administered more.

Given that there have been several documented cases of nicotine producing an inverted-U dose-response of reward and eventual aversive stimuli at higher doses, we suspect that the higher doses in males may be approaching the point at which nicotine may be aversive in this EVSA paradigm. This may be one potential explanation for the “switch” to the relationship where increased self-administration behaviors convey a decrease in mMHb intrinsic excitability.

Another interesting finding is the lack of correlations observed between PR score and mMHb neuronal excitability (Tables 1, 2). These results, or lack thereof, could suggest that excitability in the MHb is mostly modulated by behavior in the FR3 phase of intake which correlates with reinforcement-related behavior. However, it cannot be ruled out that other factors (i.e., circuits) could be at play given that modulation of subunits within the medial habenula (α5 and α3) have been shown to drastically increase nicotine intake (Fowler et al., 2011; Elayouby et al., 2021).

Though neuronal excitability correlations to self-administration behavior have rarely been used (See Cooper et al., 2023b), some studies have shown similar results in the mMHb and interesting sex-specific differences in excitability in other brain regions. First, Shih and colleagues showed an alteration in mMHb excitability and not lateral MHb excitability in response to chronic nicotine. This was demonstrated through ACh-stimulated inward currents and firing frequency after chronic nicotine administration (14 d, 1.0 mg/kg/h osmotic minipump) (Shih et al., 2015). Next, a recent study by Zhu et al. showed a nicotine-induced sex-specific difference in nucleus accumbens-projecting ventral tegmental area CRF1 dopaminergic neuron firing characteristics. Specifically, females tended to show greater tonic firing inhibition after “focal” nicotine application in an acute exposure paradigm than males. Additionally, males showed an overall greater spontaneous firing frequency as a result of acute nicotine exposure than females (Zhu et al., 2023).

The differences observed between males and females in both control baseline excitability (Fig. 1*I*) and as seen in correlations could be attributed to many factors. However, the rationale for these differences may be due to differences in nAChR populations. Prior reports show that males and females possess unique and distinct nAChR populations (Verplaetse et al., 2018; Moen and Lee, 2021). To this point, [^3^H]cytisine binding assays have verified that males exposed to nicotine will exhibit significantly greater β2-containing receptors densities than their female counterparts (Koylu et al., 1997). Further, a study by Correa and colleagues demonstrated that nicotine treatment would differentially upregulate gene expression of nAChRs within the IPN of rats (Correa et al., 2019). In the study, the researchers demonstrated via PCR that nicotine exposure would upregulate IPN α7 nAChR subunit mRNA in males and α5 nAChR subunit mRNA in females. This effect was further differentiated during nicotine withdrawal in which females showed increased α5, β3, and β4 nAChR mRNA where males showed an increase in α2, α3, β3, and β4 in the IPN (Correa et al., 2019). However, the fact that α5 nAChR subunit mRNA was shown to be upregulated in the IPN in females during nicotine intake is of particular importance as the MHb-IPN circuit contains the highest levels of α5 in the mammalian brain. Further, α5, but not β4 or β2, nAChR subunits has been demonstrated to be vital for excitability of the MHb via electrophysiological studies (Dao et al., 2014). It is important to note though, that no studies have documented sex-specific differences in nAChR populations in the MHb and further studies will be needed to elucidate differences. However, we hypothesize, due to documented sex differences in the IPN, that there are distinct differences in nAChR populations in the MHb that could possibly be driving sex differences in excitability demonstrated here.

Further, the study by Correa and colleagues demonstrated via microdialysis that females have more acetylcholine in the IPN during nicotine withdrawal than male rats (Correa et al., 2019). This fact is paired with an additional study that demonstrated an increase in GABA in females during nicotine withdrawal as compared to males and baseline levels (Carcoba et al., 2022). Given that the MHb projects almost exclusively to the IPN (Lima et al., 2017; Quina et al., 2017), these results suggest that the MHb is overactivated in females as compared to males during nicotine withdrawal. However, we believe that though the MHb is overactivated in females as compared to males in nicotine withdrawal, the circuit may behave differently during nicotine intake as demonstrated in this study. Sex-dependent differences in excitability of circuits is not unique to our study and have been observed in neighboring brain regions such as the lateral habenula (LHb) (Bell et al., 2023). In a recent study, Bell et al. demonstrated via in vivo electrophysiology that inhibition of midbrain dopaminergic neurons were sex-specifically modulated via the LHb → rostromedial tegmental nucleus pathway with males showing a significantly larger dopaminergic neuronal inhibition than females (Bell et al., 2023).

As we observed higher levels of intrinsic excitability in nicotine-naive males and observed differential modulation of the MHb based on nicotine dosage (which could possibly explain the decrease in nicotine self-administration seen in males), we believe that during the phase of nicotine intake, the MHb-IPN circuit is more excitable in males as compared to females. Our conclusion, therefore, could help explain why females tend to take in more nicotine than their male counterparts (Chaudhri et al., 2005; Flores et al., 2016).

An additional, and surprising, finding was that of sparse differences observed in rheobase and maximal spiking ability across nicotine dosage treatments observed in Figure 7. While this result may appear somewhat counterintuitive, there is ample literature to demonstrate the lack of differences in excitability metrics across conditions. Acute exposure to nicotine has been demonstrated to enhance the firing frequency and excitability of the MHb (Görlich et al., 2013; Dao et al., 2014), though it is important to emphasize the acute exposure time frame. Our paradigm though, models a chronic exposure to nicotine taking place over a series of weeks. This fact is vital given that chronic nicotine exposure in the MHb eliminates nicotine re-exposure-induced increases in firing frequency as compared to that of controls as observed in previous *ex-vivo* slice electrophysiological studies (Görlich et al., 2013). This study by Görlich and colleagues, demonstrated that only during acute nicotine exposure and during periods of nicotine withdrawal, but not chronic exposure followed by re-exposure, would ventral MHb cholinergic neuronal populations display an enhancement in firing frequency (Görlich et al., 2013). However, as observed when comparing male rheobase values, there is the obvious difference observed in 60 mg/ml nicotine plus menthol (Fig. 7*A*) as compared to every other condition. As previously discussed, the MHb has subdivisions made up of distinct nAChR populations. The medial MHb, of which our recordings are based, not only contains populations of α6 but also contains β2 and β3 (Shih et al., 2014) which commonly co-assemble to create α6β2β3* nAChRs. This fact is vital given that both nicotine and menthol are shown to upregulate and alter the stoichiometry of these nAChRs. Nicotine upregulates α6* nAChRs in the medial MHb (Henderson et al., 2014), where the addition of menthol to nicotine does not upregulate α6 as compared to nicotine alone (Henderson et al., 2017). Further, menthol alone tends to upregulate and stabilize low sensitivity α4 and α6 nAChRs being (α4)_3_(β2)_2_ and α6β2 (nonβ3), respectively (Henderson et al., 2016). However, previous studies have demonstrated that at higher doses of nicotine, α6(nonα4)β2 nAChRs downregulate and the addition of menthol to nicotine also prevents upregulation of α6(nonα4)β2 nAChRs in favor of α4β2 and α4α6β2 nAChRs (Henderson et al., 2014, 2017). For this reason, we hypothesize that the higher rheobase exhibited with males assigned to 60 mg/ml plus menthol could be due to a decrease in α6β2β3* nAChR density in the mMHb which results in a larger proportion of α3* (likely α3β4* nAChRs (Quick et al., 1999; Grady et al., 2009)).

We did not detect a link between nicotine reinforcement-related behavior and VTA dopamine neuron intrinsic excitability. This finding agrees with recent papers that demonstrated that VTA dopamine cell firing did not correlate to reward salience and NAc dopamine release (Mohebi et al., 2019; Mohebi and Berke, 2020). This work by Mohebi et al. suggested that VTA dopamine spiking may be involved in the promotion of learning; but NAc dopamine release drives motivation. In agreement with this, we did observe that female mice exhibited a significant correlation between motivation-related behavior (PR responding) and their phasic/tonic dopamine release ratio. Prior reports have shown that changes in phasic and tonic ratios are hallmarks of nicotine-induced changes in dopamine release dynamics (Rice and Cragg, 2004). The change in phasic/tonic ratios are attributed to a combination of differences in nAChR upregulation and changes in nAChR desensitization. Nicotine upregulates high-sensitivity nAChRs (including the α4_(2)_β2_(3)_ nAChR stoichiometry (Nelson et al., 2003; Nashmi et al., 2007). These high-sensitivity α4β2 nAChRs on GABA neurons are more susceptible to desensitization following acute activation (Mansvelder et al., 2002), of which the nucleus accumbens is made up of around 95% GABAergic medium spiny neurons (Robison and Nestler, 2011). Additionally, high-sensitivity α6-containing nAChRs may be upregulated. However, this may be concentration-dependent. Prior reports show that α6-containing and α4α6-containing nAChRs may upregulate at lower nicotine concentrations (Henderson et al., 2014, 2017). Higher nicotine concentrations may downregulate α6-containing (Henderson et al., 2014) or α4α6-containing nAChRs (Perez et al., 2008). Given that α6-containing and α4-containing nAChRs exhibit different desensitization and recovery kinetics in response to acute nicotine application (Henderson et al., 2016), this could be a provide a potential explanation for our observed changes in phasic/tonic ratios. Relevant to this fact, prior investigations have suggested that α6-containing nAChRs exert a higher level of control over dopamine release in the NAc when compared to the dorsal striatum (Exley et al., 2008).

We observed that male and female mice exhibited changes in phasic/tonic ratios and this is indicative of a change in the desensitization of nAChRs. Our data suggest that female mice that exhibit higher levels of PR scores also potentially exhibit greater changes in α4β2- and/or α6β2-containing nAChR upregulation and desensitization. Given our observations, this would also suggest that male mice that exhibit higher FR3 scores, may exhibit similar changes in these same nAChR populations given their significant correlation between FR3 score and phasic/tonic ratios. However, future studies utilizing single-molecule fluorescent imaging (Fu et al., 2019) will be necessary to prove upregulation of these nAChR subtypes.

The main limitation of these correlations between FSCV and EVSA lies in the fact that FSCV occurred only at one time point: after the completion of the entire EVSA paradigm in an ex vivo (slice preparation) assay. To truly capture the state of dopamine for the individual phases of the EVSA paradigm (FR3 vs PR), we will need to integrate a method such as fiber photometry in future assays. Our data also suggest that VTA dopamine intrinsic excitability does not correlate to nicotine reinforcement-related behavior but it does relate to discrimination of active and inactive nose pokes and this may suggest that the learning-associated behaviors for nicotine intake may be mediated through the VTA.

To conclude, our results show male mMHb neurons exhibit greater intrinsic excitability at baseline states when compared to females. We also show that excitability of mMHb neurons is linked to nicotine reinforcement-related behaviors; however, this is dose, sex, and flavor dependent. These data provide more support for the importance of the MHb regarding its role in nicotine dependence.

## Synthesis

Reviewing Editor: Sam Golden, The University of Washington Decisions are customarily a result of the Reviewing Editor and the peer reviewers coming together and discussing their recommendations until a consensus is reached. When revisions are invited, a fact-based synthesis statement explaining their decision and outlining what is needed to prepare a revision will be listed below. The following reviewer(s) agreed to reveal their identity: Melissa Herman. Note: If this manuscript was transferred from JNeurosci and a decision was made to accept the manuscript without peer review, a brief statement to this effect will instead be what is listed below.

Synthesis:

Dear Dr. Henderson,

Your manuscript has been reviewed by two experts, who have found the work to be a pioneering effort to identify the neural basis of nicotine/menthol self-administration with a welcomed emphasis on sex as a biological variable. The reviewers further suggest that this manuscript may provide important clinical implications for sex differences in the neural circuitry mediating motivated behavior in the context of nicotine vaping.

Based on the enthusiastic comments from the reviewers, please consider providing a point-by-point rebuttal and resubmission. Below you will find the full comments from both reviewers that must be addressedL

Reviewer 1:

The manuscript titled "Neuronal excitability in the medial habenula and ventral tegmental area is differentially modulated by nicotine dosage and menthol in a sex-specific manner" delves into the effects of nicotine and nicotine plus menthol vape self-administration, aiming to discern sex-related differences across varying nicotine dosages. The study revealed that females exhibited significantly higher self-administration rates than males. Moreover, employing patch-clamp electrophysiology, there was a direct correlation observed in males between self-administration behavior and MHb excitability under low-dose nicotine plus menthol conditions. Subsequently, the authors also explored potential correlations in the ventral tegmental area (VTA) with the amount of nicotine self-administered.

The findings indicating increased evoked excitability in male mMHb neurons compared to females are not only impressive but also highly significant. The introduction is skillfully composed, featuring a judicious choice of relevant literature that proves invaluable for comprehending the data. Conversely, the materials and methods section seem lengthy and could benefit from a more concise and schematic writing style, particularly in the final paragraph of the "E-Vape® self-administration (EVSA)" section. In summary, the manuscript provides an abundance of intriguing insights into the correlation between behavior and physiology. The following revisions are recommended to enhance clarity and facilitate the reader's comprehension and interpretation of the results:

• What is the rationale behind administering a varying number of puffs, and consequently, different intervals, during the pre-self-administration phase, as described in the "week of acclimation" text? Additionally, why is this phase not reported in the timeline in Figure 2A?

• In the "patch clamp electrophysiology" section, when referring to the control group, is this group composed of animals that received a vehicle vape, presumably PG:VG 50:50, or does it consist of a group of naïve animals?

• The discussion section of the manuscript seems well-prepared, requiring only minor revisions. Data are very intriguing. However, it's worth noting that currently, only half of the discussion is dedicated to delving into the results. In fact, the initial portion seems to consist of a sequence of results and quotes, some of which have already been introduced in the introduction section. It's only halfway through that we encounter phrases like "we suspect..." or "could suggest..." and similar expressions. I would recommend that the authors focus more on providing comprehensive explanations by establishing connections between their results and potential underlying mechanisms. This approach would not only enhance the paper's overall quality but also make it a valuable resource for other researchers seeking to develop new theoretical frameworks.

• In Figures 2F and 2G, the representation of the legend alongside the associated significance between males and females can be initially misleading. This is partly due to the y-axis extending well beyond the last point depicted.

• In Figure 4, and elsewhere, the use of only green and red to denote significance in the traces and curves can be confusing for color-blind individuals. Using easily distinguishable colors and/or adding symbols to highlight significant parameters would improve accessibility. Additionally, in Figure 4B3, there seems to be a misalignment with the p-value, with an extra "9" included.

Reviewer 2:

The current study by Olszewskiet al., investigates the impact of nicotine on excitability of medial habenula (MHb) neurons in male and female mice following self-administration of nicotine vapor at different concentration and with or without menthol flavoring. The authors report that MHb neurons from male mice displayed more current-evoked action potentials as compared to MHb neurons from female mice at baseline. Female mice displayed increased self-administration of nicotine at the lower concentration and nicotine + menthol at both lower and higher concentrations. Self-administration behavior was not significantly correlated with MHb excitability measures in females with the exception of a negative correlation between rheobase and FR3 score in the higher concentration nicotine + menthol group. Male mice self-administered less nicotine and nicotine + menthol than females but male MHb excitability was significantly correlated with FR3 self-administration behavior in several measures of excitability including whole positive correlations with cell firing and maximum spiking in the low concentration nicotine + menthol group, positive correlations between rheobase and FR3 score in both high concentration nicotine alone and nicotine + menthol groups, and negative correlations with maximum spiking and FR3 score in both high concentration nicotine alone and nicotine + menthol groups. Additionally, α6 subunit ventral tegmental area (VTA) neurons from male and female GFP-α6 mice subjected to the same nicotine vapor self-administration paradigm displayed positive correlations between rheobase and active: inactive nosepoke responding and positive correlations between the ratio of phasic:tonic DA release in the Nucleus Accumbens and FR3 score in males and between progressive ratio (PR) in females.

There are some minor issues with the communication of the findings that should be addressed, but overall this is a well-designed study with important clinical implications for sex differences in the neural circuitry mediating motivated behavior in the context of nicotine vaping.

1. The Methods section describes a 5 day period of non-contingent exposure prior to EVSA that uses different exposure times for different nicotine concentrations ("6 mg/mL {plus minus} menthol received a vapor delivery every 4 minutes and 48 seconds which totaled 25 vapor deliveries over two hours...60 mg/mL nicotine {plus minus} menthol received a vapor delivery every 12 minutes totaling 10 vapor deliveries in two hours") Some rationale and justification for this difference in non-contingent acclimation period should be provided.

2. The rheobase of male and female MHb neurons shown in Figure 1H looks like a trend for increased rheobase in female MHb neurons. While the statistics reported for this do not reflect a significant change the trend is visibly apparent. Authors may wish to consider this in more detail with additional recordings or with a note in the text.

3. The 5 days of non-contingent exposure to nicotine vapor should be included in the experimental timeline in Figure 2A.

4. How long after the EVSA session were slices prepared for electrophysiological recording? Depending on the last vapor delivery that was received prior to end of the EVSA session, mice could have very different serum nicotine levels, which may influence excitability. If possible, authors should report the time from last vapor delivery between groups or at least consider this in their reporting of the Results.

5. Authors justify the inclusion of mice even if the previously-published standard of a 2:1 active-to-inactive nose-poke distinction was not achieved. This is warranted given the interest in examining mice with a rage of self-administration responding, however it may be worth noting the overall discrimination ratios of both males and females as this could be useful in data interpretation.

6. Authors should provide more detail on the experimental endpoint of Active vs Inactive Ratio that is reported in Figure 7C3. This is explained in more detail in the Discussion as active vs inactive nosepoke ratio but clarification in the Results would improve overall data communication.

7. The self-administration behavior for the male and female GFP-α6 mice should be included in text or in a Figure. Were the sex differences in self-administration behavior seen in the C57BL/6J mice also observed in the GFP-α6 mice? There doesn't seem to be much difference in FR3 scores but the scores do seem higher overall. This should be noted.

8. In Figure 7, it is unclear if the representative traces shown in B1-B4 come from male or female mice. Figure 7 could benefit from additional labeling for nicotine and nicotine + menthol labeling as well, particularly as experimental groups are different from what was presented in previous figures and nicotine concentration differed by sex.

9. It would be good to report (and potentially include in Figures, if notable) the direct comparisons of excitability measures between groups within each sex, not just in correlations with self-administration behavior.

10. The fast scan cyclic voltammetry data are not mentioned in the Abstract and only superficially considered in the Discussion. Some additional detail would be useful.

Minor Comments:

- There are two references to Fig 1E in the first paragraph of the Results section, it seems as though the second should actually refer to Figure 1I

- The repeated use of 'ns' for nonsignificant findings is distracting and the authors may wish to remove them unless they are more relevant (i.e. data look like they may be significant).

- The use of A1-A4 and B1-B4 (instead of standard A, B, C, D, etc...) in Figures 3-7 is inconsistent with other figures. Standardizing figure format would be useful.

- Standardizing color for significant and non-significant correlations would also improve consistency

## Author Response

We sincerely thank the reviewers for their input and comments on our manuscript entitled, "Neuronal excitability in the medial habenula and ventral tegmental area is differentially modulated by nicotine dosage and menthol in a sex-specific manner". We believe that the concerns and comments from the reviewers have helped to greatly strengthen our work and that the quality and clarity of the manuscript has been greatly improved. Below are our responses to the comments of the reviewers.

Reviewer 1:

• What is the rationale behind administering a varying number of puffs, and consequently, different intervals, during the pre-self-administration phase, as described in the "week of acclimation" text? Additionally, why is this phase not reported in the timeline in Figure 2A?

Response: The rationale behind the varying times of air puffs is to avoid toxic levels specifically at the high doses of nicotine. This has now been added in text for clarity.

• In the "patch clamp electrophysiology" section, when referring to the control group, is this group composed of animals that received a vehicle vape, presumably PG:VG 50:50, or does it consist of a group of naïve animals?

Response: In these experiments, the control group is composed of nicotine naïve animals. We have added this detail into the in-text session for clarity.

• The discussion section of the manuscript seems well-prepared, requiring only minor revisions. Data are very intriguing. However, it's worth noting that currently, only half of the discussion is dedicated to delving into the results. In fact, the initial portion seems to consist of a sequence of results and quotes, some of which have already been introduced in the introduction section. It's only halfway through that we encounter phrases like "we suspect..." or "could suggest..." and similar expressions. I would recommend that the authors focus more on providing comprehensive explanations by establishing connections between their results and potential underlying mechanisms. This approach would not only enhance the paper's overall quality but also make it a valuable resource for other researchers seeking to develop new theoretical frameworks.

Response: We appreciate this comment and agree that the discussion needed to be worked on. To address the concern, we have added additional texts delving into sex differences in nAChR populations and circuit excitability and made a connection to the presented data for interpretation.

• In Figures 2F and 2G, the representation of the legend alongside the associated significance between males and females can be initially misleading. This is partly due to the y-axis extending well beyond the last point depicted.

Response: We agree that how the legend was presented could be confusing for readers. These figures have been amended with the legend now moved to besides the data.

• In Figure 4, and elsewhere, the use of only green and red to denote significance in the traces and curves can be confusing for color-blind individuals. Using easily distinguishable colors and/or adding symbols to highlight significant parameters would improve accessibility. Additionally, in Figure 4B3, there seems to be a misalignment with the p-value, with an extra "9" included.

Response: We have replaced our red/green significance highlights to red/blue as those are more easily distinguished among color-blind individuals. We have also added asterisks to highlight significant differences.

Reviewer 2:

1. The Methods section describes a 5 day period of non-contingent exposure prior to EVSA that uses different exposure times for different nicotine concentrations ("6 mg/mL {plus minus} menthol received a vapor delivery every 4 minutes and 48 seconds which totaled 25 vapor deliveries over two hours...60 mg/mL nicotine {plus minus} menthol received a vapor delivery every 12 minutes totaling 10 vapor deliveries in two hours") Some rationale and justification for this difference in non-contingent acclimation period should be provided.

Response: We agree with the reviewer's comment and have added justification for the time periods in the passive session in text.

2. The rheobase of male and female MHb neurons shown in Figure 1H looks like a trend for increased rheobase in female MHb neurons. While the statistics reported for this do not reflect a significant change the trend is visibly apparent. Authors may wish to consider this in more detail with additional recordings or with a note in the text.

Response: we hesitate to overinterpret our data. We do agree that there is a strong trend and we have acknowledged this but also reiterate that this is not significantly different (p = 0.08, unpaired t-test).

3. The 5 days of non-contingent exposure to nicotine vapor should be included in the experimental timeline in Figure 2A.

Response: This has been amended and is now in Figure 2A.

4. How long after the EVSA session were slices prepared for electrophysiological recording? Depending on the last vapor delivery that was received prior to end of the EVSA session, mice could have very different serum nicotine levels, which may influence excitability. If possible, authors should report the time from last vapor delivery between groups or at least consider this in their reporting of the Results.

Response: We have added this information into the electrophysiological methods portion of the paper. Mice were utilized for electrophysiology no more than 24 hours after their final EVSA session.

5. Authors justify the inclusion of mice even if the previously-published standard of a 2:1 active-to-inactive nose-poke distinction was not achieved. This is warranted given the interest in examining mice with a rage of self-administration responding, however it may be worth noting the overall discrimination ratios of both males and females as this could be useful in data interpretation.

Response: We agree with the reviewer and have now noted the overall pass rate of our active: inactive nose poke criteria with males and females for clarification.

6. Authors should provide more detail on the experimental endpoint of Active vs Inactive Ratio that is reported in Figure 7C3. This is explained in more detail in the Discussion as active vs inactive nosepoke ratio but clarification in the Results would improve overall data communication.

Response: We have expanded the results section that presents the correlation between VTA dopamine neuron excitability and active: inactive ratio. Here we took the mean of the A:I ratio for the FR3 sessions (EVSA sessions 11 - 15). Accordingly, this is the same period in which we calculate the FR3 score.

7. The self-administration behavior for the male and female GFP-α6 mice should be included in text or in a Figure. Were the sex differences in self-administration behavior seen in the C57BL/6J mice also observed in the GFP-α6 mice? There doesn't seem to be much difference in FR3 scores but the scores do seem higher overall. This should be noted.

Response: Unpublished data from our laboratory has demonstrated that there is no difference in self-administration based on the genotype of our mice. For that reason, we have not included any separate data for analysis.

8. In Figure 7, it is unclear if the representative traces shown in B1-B4 come from male or female mice. Figure 7 could benefit from additional labeling for nicotine and nicotine + menthol labeling as well, particularly as experimental groups are different from what was presented in previous figures and nicotine concentration differed by sex.

Response: We apologize for the confusion in presenting this data. Previously, all representative recordings in the previous Figure 7 were from male mice. We now include current clamp recordings for male and female mice as with all other figures in this manuscript. We have also clearly labeled the different nicotine doses used in male (6 mg/mL + menthol) and female (60 mg/mL + menthol) mice.

9. It would be good to report (and potentially include in Figures, if notable) the direct comparisons of excitability measures between groups within each sex, not just in correlations with self-administration behavior.

Response: We agree that more direct comparisons, as mentioned, would be helpful for our results. We have added a new figure (Figure 7 A-D) to address this concern.

10. The fast scan cyclic voltammetry data are not mentioned in the Abstract and only superficially considered in the Discussion. Some additional detail would be useful.

Response: We now include a brief summary of our FSCV findings in the abstract and have now expanded our discussion to include considerations for the FSCV observations.

Minor comments:

- There are two references to Fig 1E in the first paragraph of the Results section, it seems as though the second should actually refer to Figure 1I

Response: We have amended this error.

- The repeated use of 'ns' for nonsignificant findings is distracting and the authors may wish to remove them unless they are more relevant (i.e. data look like they may be significant).

Response: We have taken out the "NS" notation as recommended except in places that look as though they are trending towards significance.

- The use of A1-A4 and B1-B4 (instead of standard A, B, C, D, etc...) in Figures 3-7 is inconsistent with other figures. Standardizing figure format would be useful.

Response: We have amended this and standardized so that every figure and its panels now follow the A, B, C, etc. format.

- Standardizing color for significant and non-significant correlations would also improve consistency

Response: We have standardized the color format and now all significance and non-significance is of a red-blue color scheme.
